# Tests of chameleon gravity

**DOI:** 10.1007/s41114-018-0011-x

**Published:** 2018-03-16

**Authors:** Clare Burrage, Jeremy Sakstein

**Affiliations:** 1 School of Physics and Astronomy, University of Nottingham, Nottingham, NG7 2RD UK; 2 Department of Physics and Astronomy, Center for Particle Cosmology, University of Pennsylvania, Philadelphia, PA 19104 USA

**Keywords:** Scalar-tensor theories, Modified gravity, Tests of gravity

## Abstract

Theories of modified gravity, where light scalars with non-trivial self-interactions and non-minimal couplings to matter—chameleon and symmetron theories—dynamically suppress deviations from general relativity in the solar system. On other scales, the environmental nature of the screening means that such scalars may be relevant. The highly-nonlinear nature of screening mechanisms means that they evade classical fifth-force searches, and there has been an intense effort towards designing new and novel tests to probe them, both in the laboratory and using astrophysical objects, and by reinterpreting existing datasets. The results of these searches are often presented using different parametrizations, which can make it difficult to compare constraints coming from different probes. The purpose of this review is to summarize the present state-of-the-art searches for screened scalars coupled to matter, and to translate the current bounds into a single parametrization to survey the state of the models. Presently, commonly studied chameleon models are well-constrained but less commonly studied models have large regions of parameter space that are still viable. Symmetron models are constrained well by astrophysical and laboratory tests, but there is a desert separating the two scales where the model is unconstrained. The coupling of chameleons to photons is tightly constrained but the symmetron coupling has yet to be explored. We also summarize the current bounds on *f*(*R*) models that exhibit the chameleon mechanism (Hu and Sawicki models). The simplest of these are well constrained by astrophysical probes, but there are currently few reported bounds for theories with higher powers of *R*. The review ends by discussing the future prospects for constraining screened modified gravity models further using upcoming and planned experiments.

## Introduction

Since its publication in 1915, Einstein’s theory of general relativity (GR) has withstood the barrage of observational tests that have been thrown at it over the last century. From Eddington’s pioneering measurement of light bending by the Sun in 1919 to the first detection of gravitational waves by the LIGO/Virgo consortium in 2015 (Abbott et al. 2016a, b), its predictions have been perfectly consistent with our observations. To test the predictions of any theory requires alternatives with differing predictions and, for this reason, alternative theories of gravity have a history that is almost as rich and varied as that of GR itself.

The zoo of modified gravity theories is both vast and diverse (see Clifton et al. 2012; Joyce et al. 2015; Koyama 2016; Bull et al. 2016, for some compendia of popular models) but all have one thing in common: they break one of the underlying assumptions of general relativity. From a theoretical standpoint, GR is the unique low-energy theory of a Lorentz-invariant massless spin-2 particle (Weinberg 1965), and any modification must necessarily break one of these assumptions. Several interesting and viable Lorentz-violating theories exist that may have some insight for the quantum gravity problem (Blas and Lim 2015), and, similarly, healthy theories of massive spin-2 particles have recently been constructed (de Rham 2014).

An alternative to these approaches is to introduce new fields that couple to gravity. One of the simplest possible options is to include a new scalar degree of freedom. These scalar–tensor theories of gravity are particularly prevalent, and are natural extensions of general relativity. Scalars coupled to gravity appear in many UV completions of GR such as string theory and other higher-dimensional models, but the cosmological constant problem and the nature of dark energy, two modern mysteries that GR alone cannot account for, are driving a vigorous research effort into infra-red scalar–tensor theories, with much of the effort focussing on light scalars (with cosmologically relevant masses) coupled to gravity.

Typically, the existence of such scalars are in tension with experimental bounds. If the scalar is massless, or has a Compton wavelength larger than the size of the solar system (which is certainly the case for Hubble-scale scalars), the theory’s predictions typically fall within the remit of the parameterized post-Newtonian (PPN) formalism for testing gravity in the solar system (see Will 2004, and references therein). Scalars whose Compton wavelengths are smaller than $\sim$ AU predict deviations from the inverse-square law inside the solar system, which has been tested on interplanetary scales using lunar laser ranging (LLR) (Williams et al. 2004), and down to distances of $\mathcal {O}(\upmu \mathrm {m})$ using laboratory-based experiments such as the Eöt-Wash torsion balance experiment (Adelberger et al. 2003). In many cases, scalar–tensor theories spontaneously break the equivalence principle so that objects of identical mass but differing internal compositions fall at different rates in an external gravitational field. This too can be tested with LLR and terrestrial searches.

Recently, the simultaneous observation of gravitational waves and a gamma ray burst from a binary neutron star merger (GW170817 and GRB 170817A) (Abbott et al. 2017a, b) by the LIGO/Virgo collaboration and the Fermi and INTEGRAL satellites has placed a new and stringent bound on modified gravity theories. The close arrival time of the gravitational wave and photon signal ($\delta t<1.7\mathrm {\ s}$) constrains the relative difference speed of photons (*c*) and gravitons ($c_T$) to be close to unity at the $10^{-15}$ level ($-3\,\times \,10^{-15}<|c_T^2-c^2|/c^2<7\,\times \,10^{-16}$) (Sakstein and Jain 2017; Ezquiaga and Zumalacárregui 2017; Creminelli and Vernizzi 2017; Baker et al. 2017; Crisostomi and Koyama 2018; Langlois et al. 2017; Dima and Vernizzi 2017; Bartolo et al. 2017), where the upper and lower bounds correspond to a $\sim 10$ s uncertainty in the time between the emission of the photons and the emission of the gravitational waves Abbott et al. (2017b). Many scalar–tensor theories predict that the difference between the speeds of gravitons and photons is of order unity for models that act as dark energy (Bellini and Sawicki 2014; Brax et al. 2016) and so this bound represents a new hurdle for them to overcome.

These stringent bounds imply that the simplest theories with light scalars have couplings to matter that must be irrelevant on cosmological scales. Theories that try to avoid this problem using a large mass to pass solar system tests must have a Compton wavelength $\le \mathcal {O}(\upmu \mathrm {m})$, in which case they too are cosmologically inconsequential. Ostensibly, it seems that scalar–tensor theories are trivial in a cosmological setting, but the link between solar system tests of gravity and cosmological scalar–tensor theories can be broken. Indeed, the last decade of scalar–tensor research can aptly be epitomized by two words: *screening mechanisms*.

Screening mechanisms utilize non-linear dynamics to effectively decouple solar system and cosmological scales. At the heart of screening mechanisms lies the fact that there are 29 orders-of-magnitude separating the cosmological and terrestrial densities and 20 orders of magnitude separating their distance scales. As a result, the properties of the scalar can vary wildly in different environments. The quintessential example of a screening mechanism being used to ensure a dark energy scalar avoids solar system constraints is the chameleon mechanism (Khoury and Weltman 2004a, b; earlier predecessors include Gessner 1992; Pietroni 2005; Olive and Pospelov 2008). In chameleon models, the mass of the scalar is an increasing function of the ambient density. This allows it to have a sub-micron Compton wavelength in the solar system but be light on cosmological scales. Later, a closely related second dark energy screening mechanism was discovered: the symmetron mechanism (Hinterbichler and Khoury 2010; Hinterbichler et al. 2011a). Earlier work had studied a similar model but with a different motivation (Pietroni 2005; Olive and Pospelov 2008), and string-inspired models with similar phenomenology have also been proposed (Damour and Polyakov 1994; Brax et al. 2011a). Unlike the chameleon, the symmetron has a light mass on all scales and instead screens by driving its coupling to matter to zero when the density exceeds a certain threshold. A third mechanism, the environment-dependent dilaton was subsequently discovered that screens in a similar manner (Brax et al. 2010a).

In this work, we will only discuss screening mechanisms of this type, which rely on non-linear self-interaction terms in the potential. A final class of screening, which relies on non-linearities in the kinetic sector screen through what is known as the Vainshtein mechanism (Babichev and Deffayet 2013; Joyce et al. 2015). These theories will not be discussed here as the phenomenology of these models, and therefore the most constraining observables, are very different to that of the chameleon and symmetron models. Similarly, we will not discuss massive gravity (de Rham et al. 2011; Hinterbichler 2012; de Rham 2014; de Rham et al. 2017), which screens using the Vainshtein mechanism, for the same reason. We note, however, that many models that do screen using the Vainshtein mechanism (as well as those that predict a mass in the graviton dispersion relation such as massive gravity) are severely constrained by the new bounds from the observation of gravitational waves and photons from GW170817/GRB 170817A discussed above if they are to simultaneously act as dark energy (Sakstein and Jain 2017; Baker et al. 2017; Ezquiaga and Zumalacárregui 2017; Creminelli and Vernizzi 2017; Crisostomi and Koyama 2018; Langlois et al. 2017). [In the case of massive gravity, solar system tests are stronger than the LIGO/Fermi bound (Baker et al. 2017)]. The models we will discuss in this review (chameleon/symmetron/dilaton) predict that $c_T=c$ identically and so this bound is irrelevant for them.

Scalar fields with screening mechanisms cannot simultaneously screen and self-accelerate cosmologically (Wang et al. 2012), but they can act as a dark energy quintessence field (Copeland et al. 2006), i.e., they require a cosmological constant term to drive the cosmic acceleration and they are capable of producing deviations from GR on linear and non-linear cosmological scales as well as astrophysical scales (see Jain et al. 2013b; Sakstein 2014a, and references therein). In addition to this, many candidate UV completions of GR such as string theory predict a multitude of scalars that couple to matter and screening mechanisms are a convenient method of hiding such additional degrees of freedom. For these reasons, screening mechanisms are considered interesting and novel paragon for alternative theories of gravity and, as such, there is an ongoing experimental search for screened scalars. Being designed to evade conventional tests of gravity, screening mechanisms have inspired novel and inventive approaches to search for them experimentally. These range from reinterpreting the results of experiments not designed to look for them, to designing instruments specifically adapted to testing their unique properties, to using astrophysical objects that have never before been used to test gravity, such as Cepheid stars and galaxy clusters. In many cases, new and imaginative scenarios have been concocted.

These searches typically use different parametrizations, making them difficult to compare with one another. The purpose of this review is to collect the state-of-the-art constraints coming from laboratory and astrophysical tests, and to combine them into a single parametrization. This not only makes it clear which models are ruled out by different experiments, but also aides in deciding the optimum search strategy for exploring the remaining models. In many cases, we will extend the experimental results to models to which they have not previously been applied.

This review is organized as follows. In Sect. 2, we will introduce the different screening mechanisms we will consider in this review, outline their salient features, and present the parameters we will use to compare constraints. In Sect. 3, we will discuss how screening works in both astrophysical and laboratory settings. Section 4 contains a brief description of the experiments that have been used to constrain screening mechanisms, and translates the constraints into our parametrization. The crux of this review is presented in Sect. 5, where we combine all of the contemporary constraints from various experiments into a series of diagrams that show which regions of parameter space are ruled out, and how different experiments compare in the same parametrization. We do this for chameleon and symmetron modes. In Sect. 6, we conclude by discussing the implications of the constraints for screened modified gravity, and future prospects for constraining the remaining parameter space.

## Screening mechanisms

The screening mechanisms that we consider in this review are all specific subsets of the general scalar–tensor theory

2.1 $$\begin{aligned} S=\int \, \mathrm{d}^4 x\sqrt{-g}\left[ \frac{R}{16\pi G}-\frac{1}{2}\nabla _\mu \phi \nabla ^\mu \phi -V(\phi )\right] +S{_\mathrm{m}}[\tilde{g}_{\mu \nu },\phi ], \end{aligned}$$

which describes a canonically normalised scalar field $\phi$ subject to a potential $V(\phi )$ and conformally (Weyl) coupled to matter through the *Jordan frame metric*

2.2 $$\begin{aligned} \tilde{g}_{\mu \nu }=A^2(\phi )g_{\mu \nu }. \end{aligned}$$

It is this non-minimal coupling described by the *coupling function* $A(\phi )$ that results in deviations from GR.^1^ In particular, the *Einstein frame metric*, $g_{\mu \nu }$, is a solution of Einstein’s equations sourced by both matter and the scalar stress energy tensors, but matter moves on geodesics of the Jordan frame metric, $\tilde{g}_{\mu \nu }$. In what follows, we work only with the Einstein frame version of the theory. Classically, all observable quantities will be independent of the choice of frame and our choice of the Einstein frame is purely for calculational convenience. In the Jordan frame there would be no direct coupling between the scalar fields and matter, but instead the gravitational action will depend non-trivially on the scalar field. In this frame matter, particles travel on geodesics of the Jordan frame metric, but the evolution of the gravitational potentials is modified by their mixing with the scalar field.

As an example of motion in the Einstein frame, consider a non-relativistic particle in the Newtonian limit. This particle moves on geodesics of $\tilde{g}_{\mu \nu }$ and so, defining the tensor ${\mathcal {K}}^\alpha _{\mu \nu }\equiv \tilde{\varGamma }^\alpha _{\mu \nu }-\varGamma ^\alpha _{\mu \nu }$, the Newtonian limit of the geodesic equation is (Sakstein 2014a; Burrage and Sakstein 2016)

2.3 $$\begin{aligned} \ddot{x}^i+\varGamma ^i_{00}=-\kappa ^i_{00}=-\frac{\beta (\phi )}{{M_\mathrm{{pl}}}}\nabla ^i\phi , \end{aligned}$$

where a dot denotes a derivative with respect to proper time and we have calculated ${\mathcal {K}}^i_{00}$ using (2.2) (see Wald 2010; Zumalacárregui and García-Bellido 2014). The *coupling* is

2.4 $$\begin{aligned} \beta (\phi )\equiv {M_\mathrm{{pl}}}\frac{\, \mathrm{d}\ln A}{\, \mathrm{d}\phi }. \end{aligned}$$

The Christoffel symbol $\varGamma ^i_{00}=\partial ^i\varPhi _\mathrm{N}$ contains the Newtonian force and so we can interpret

2.5 $$\begin{aligned} F_5=-\frac{\beta (\phi )}{{M_\mathrm{{pl}}}}\nabla \phi \end{aligned}$$

as a new or *fifth-*force. In this review, we do not consider scalars with non-minimal kinetic terms which screen through the Vainshtein mechanism.

Another important consequence of the coupling to matter is that the field is sourced by the trace of the energy-momentum tensor so that its equation of motion is

2.6 $$\begin{aligned} \Box \phi =\frac{\, \mathrm{d}V(\phi )}{\, \mathrm{d}\phi }-\frac{\beta (\phi )T}{{M_\mathrm{{pl}}}}. \end{aligned}$$

Note that $T=g_{\mu \nu }T^{\mu \nu }$ where $T^{\mu \nu }=2/\sqrt{-g}\delta S{_\mathrm{m}}/\delta g_{\mu \nu }$ is the Einstein frame energy-momentum tensor. This is not covariantly conserved ($\nabla _\mu T^{\mu \nu }\ne 0$) because matter moves on geodesics of $\tilde{g}$; it is the Jordan frame metric $\tilde{T}^{\mu \nu }=2/\sqrt{-\tilde{g}}\delta S{_\mathrm{m}}/\delta \tilde{g}_{\mu \nu }$ that satisfies $\tilde{\nabla }_\mu \tilde{T}^{\mu \nu }=0$. The two are related via $T^{\mu \nu }=A^6\tilde{T}^{\mu \nu }$ (Wald 2010; Sakstein 2014a). For non-relativistic matter, one has^2^ $T=-\rho \approx -\tilde{\rho }\approx \tilde{T}$, where we have ignored post-Newtonian terms (Hui et al. 2009; Sakstein 2014b). The equation of motion is then

2.7 $$\begin{aligned} \Box \phi =\frac{\, \mathrm{d}V(\phi )}{\, \mathrm{d}\phi }+\frac{\beta (\phi )\rho }{{M_\mathrm{{pl}}}}=\frac{\, \mathrm{d}V_\mathrm{eff}}{\, \mathrm{d}\phi }, \end{aligned}$$

which defines a density-dependent effective potential^3^

2.8 $$\begin{aligned} V_\mathrm{eff}(\phi )=V(\phi )+\rho \ln A(\phi ). \end{aligned}$$

It is this that governs they dynamics of $\phi$ and not $V(\phi )$ solely.

In order to classify different screening mechanisms it is instructive to consider the field profile sourced by a spherical object of mass ${\mathcal {M}}$ and radius ${\mathcal {R}}$ embedded in a medium of background density $\rho _0$. If the effective potential has a minimum then the field in this medium will assume the value $\phi _0=\phi _\mathrm{min}(\rho _0)$ where this is achieved. Expanding the field about this background value $\phi =\phi _0+\delta \phi$, where $\delta \phi$ is the field sourced by the point mass, and $\phi _0$ the uniform background value (i.e. we have performed a background-object split), we have the equation of motion for a massive scalar

2.9 $$\begin{aligned} \nabla ^2\delta \phi -m_\mathrm{eff}^2(\phi _0)\delta \phi =\frac{\beta (\phi _0)\rho (r)}{{M_\mathrm{{pl}}}}, \end{aligned}$$

where the effective mass

2.10 $$\begin{aligned} m_\mathrm{eff}^2(\phi )\equiv V_\mathrm{eff}''(\phi ) \end{aligned}$$

is the mass of small fluctuations about the minimum of the effective potential. The scalar potential outside the source is then

2.11 $$\begin{aligned} \delta \phi =\frac{\beta (\phi _0)}{4\pi {M_\mathrm{{pl}}}}\frac{f({\mathcal {M}},{\mathcal {R}})}{r}e^{-m_\mathrm{eff}r}, \end{aligned}$$

where the undetermined function $f({\mathcal {M}},{\mathcal {R}})$ is a model dependent function of the source mass parameters. When the source is a point mass one simply has $f({\mathcal {M}},{\mathcal {R}})={\mathcal {M}}$ but in general the effective mass may vary inside the object and the object may have a non-trivial density profile. From Eq. (2.11), it is clear that there are three ways one can suppress the effects of the scalar. Either

1. The mass $m_\mathrm{eff}r\gg 1$ so that the force is short ranged,
2. The coupling to matter $\beta (\phi _0)\ll 1$, or
3. Not all of the mass sources the scalar field.

Of course, one could simply choose the parameters such that either of the first two conditions is satisfied but this leads to a trivial situation where the modifications of gravity are negligible on all scales, and requires fine-tuning the parameters. We are interested in theories where solar system tests are satisfied trivially but strong modifications may appear on other scales, producing new and interesting phenomena that may be relevant to small-scale physics or dark energy and cosmology. Said another way, we would like to construct theories that exhibit some environmental dependence of the screening, for example so that conditions 1 or 2 are only satisfied locally. The density-dependence of the effective potential aids us here because the ambient density of different objects can vary over many orders of magnitude. For example, there are 29 orders of magnitude separating the mean cosmic density from the density on Earth. The essence of screening mechanisms is that the effective potential is chosen such that the minimum is density-dependent so that the field can assume different values in different environments so that the scalar potential can be dynamically suppressed.

It is possible to construct models with the requisite density-dependent minimum such that one or more of the conditions above are satisfied. Models that utilize a combination of the first and third condition are typically known as *chameleon* models^4^ (Khoury and Weltman 2004a) and models that utilize the second are known as either symmetron (Hinterbichler and Khoury 2010) or dilaton models (Brax et al. 2010a).

### Chameleon screening

As remarked above, the chameleon is constructed to give an effective mass that increases with the density. A wide variety of potentials and coupling functions can achieve this; here we follow the existing literature and focus on power-law potentials and exponential couplings,

2.12 $$\begin{aligned} V(\phi )=\frac{\varLambda ^{n+4}}{\phi ^n},\quad A(\phi )=e^{\frac{\phi }{M_\mathrm{c}}}, \end{aligned}$$

so that the effective potential is then

2.13 $$\begin{aligned} V_\mathrm{eff}(\phi )=\frac{\varLambda ^{n+4}}{\phi ^n}+\rho \frac{\phi }{M_\mathrm{c}}, \end{aligned}$$

where $M_c = M_P/\beta$. Theories with $M_c\sim M_P$, $\beta \sim 1$ have gravitational strength couplings to matter. The effective potential has a density-dependent minimum given by

2.14 $$\begin{aligned} \phi _\mathrm{min}(\rho )=\left( \frac{nM_\mathrm{c}\varLambda ^{4+n}}{\rho }\right) ^{\frac{1}{n+1}}. \end{aligned}$$

The effective mass about this minimum is

2.15 $$\begin{aligned} m_\mathrm{eff}^2=V''_\mathrm{eff}(\phi )=n(n+1)\varLambda ^{n+4}\left( \frac{\rho }{nM_\mathrm{c}\varLambda ^{n+4}}\right) ^{\frac{n+2}{n+1}}. \end{aligned}$$

For $n>-1$ this certainly satisfies our requirement that the mass is an increasing function of the density, with the exception of $n=0$, which does not admit a minimum. Negative even integers, i.e., $n=-4,-6,-8,\ldots$ also have this property with the exceptions $n=-1,\,-2$, which do not allow the mass to vary with the density. There is no minimum when $n=-3,-5,-7,\ldots$ and so there are no viable chameleon mechanism in these cases.

**Fig. 1:**
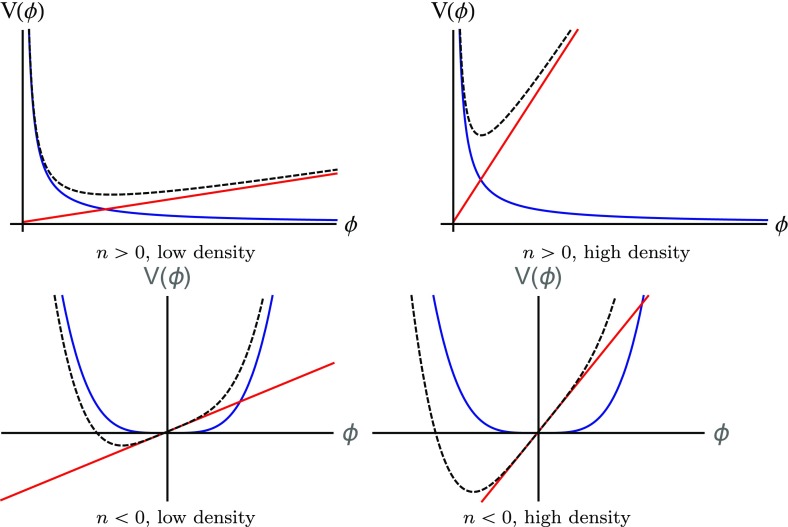
Sketches of the chameleon effective potential for positive *n* (upper panels) and negative *n* (lower panels). The left and right panels show the cases of low and high density environments respectively. The blue lines show the bare potential and the red lines show the contribution from the coupling to matter. The black dashed lines show the resultant effective potential, which is the sum of the red and blue lines, and governs the dynamics of the scalar

The chameleon mechanism is illustrated in Fig. 1, which sketches the effective potential, as well as the separate contributions from the bare potential and the matter coupling, for positive and negative *n* in both high and low densities. One can clearly see that the curvature around the high-density minimum is larger than around the low-density minimum, implying a larger mass for fluctuations. In practice, the difference can be several orders of magnitude, giving rise to very efficient screening.

Since chameleon models are unable to self-accelerate cosmologically (Wang et al. 2012), one typically adds a cosmological constant to the bare potential in order to account for dark energy. In this case, one has

2.16 $$\begin{aligned} V(\phi )= \varLambda _\mathrm{DE}^4 + \frac{\varLambda ^{n+4}}{\phi ^n} \end{aligned}$$

with $\varLambda _\mathrm{DE}=2.4$ meV. A common origin for the cosmological constant and the chameleon is an enticing scenario, for example one could have $V(\phi )=\varLambda ^4\exp (\varLambda ^4/\phi ^n)$ (Brax et al. 2004b), and so special attention is often paid to the case $\varLambda =\varLambda _\mathrm{DE}=2.4$ meV.

Another important model is the case $n=-4$. In this case, the mass-scale $\varLambda$ that governs that chameleon self-interactions is absent and one instead has the renormalisable potential

2.17 $$\begin{aligned} V(\phi )=\varLambda _\mathrm{DE}^4+\lambda _\mathrm{c}\phi ^4. \end{aligned}$$

One generally expects $\lambda _\mathrm{c}\sim \mathcal {O}(1)$ to be natural since values larger than this can give rise to large quantum corrections to the potential and smaller values typically require some degree of fine-tuning. Comparing with the form of the potential when $n\ne 4$ one has $\lambda _\mathrm{c}=(\varLambda /\varLambda _\mathrm{DE})^4$. Even with this choice of renormalisable potential, the full chameleon model itself is non-renormalisable because the coupling to matter introduces higher-order operators of the form

2.18 $$\begin{aligned} {\mathcal {L}}\supset T\ln [A(\phi )]\sim \left( \frac{\phi }{M_\mathrm{c}}+\mathcal {O}{\left( \frac{\phi ^2}{M_\mathrm{c}^2}\right) }+\cdots \right) T. \end{aligned}$$

We will discuss this further below.

#### *f*(*R*) models

Theories of gravity where one replaces the Einstein–Hilbert action by a generic function *R*, known as *f*(*R*) theories (see De Felice and Tsujikawa 2010, for more general reviews), can screen using the chameleon mechanism, indeed they need to possess a form of screening mechanism to be compatible with solar system constraints. The first example of such a model was that of Hu and Sawicki (2007)

2.19 $$\begin{aligned} S=\frac{1}{16\pi G}\int \, \mathrm{d}^4x\sqrt{-\tilde{g}}\left( {R+f(R)}\right) +S_\mathrm{m}[\tilde{g}];\quad f(R)=-a\frac{\mu ^2}{1+(R/\mu ^2)^{-b}},\nonumber \\ \end{aligned}$$

where *a* and *b* and both positive and $R=R(\tilde{g})$. Expanding this action for high curvatures ($R\gg \mu ^2$) one finds that

2.20 $$\begin{aligned} f(R)=-a\mu ^2+a\mu ^2\left( \frac{R}{\mu ^2}\right) ^{-b}+\cdots \end{aligned}$$

so that the theory looks like a cosmological constant with small corrections to GR. Indeed, one can tune the constants *a* and *b* to match with the $\varLambda$CDM cosmological model and one is left with small deviations from GR at the level of cosmological perturbations. In the low-curvature regime ($R\ll \mu ^2$) the theory behaves like inverse-power law models where $f(R)\sim (R/\mu )^{-b}$ so that deviations from GR are suppressed. One can see the chameleon mechanism in action using the equivalence between *f*(*R*) and scalar–tensor theories (Chiba 2003). Introducing the auxiliary field $\varphi$, a classically-equivalent action to (2.19) is

2.21 $$\begin{aligned} S=\frac{1}{16\pi G}\int \, \mathrm{d}^4x\sqrt{-\tilde{g}}\left( R+f(\varphi )+\frac{\, \mathrm{d}f}{\, \mathrm{d}\varphi }\left[ R-\varphi \right] \right) +S_\mathrm{m}[\tilde{g}]. \end{aligned}$$

One can verify this by varying with respect to $\varphi$ to find $\varphi =R$ on shell, thereby recovering the action (2.19). Introducing the Weyl-rescaled Einstein frame metric

2.22 $$\begin{aligned} \tilde{g}_{\mu \nu }=A^2(\phi )g_{\mu \nu };\quad A^2(\varphi )=1+\frac{\, \mathrm{d}f}{\, \mathrm{d}\varphi }, \end{aligned}$$

the action (2.21) can be recast into a scalar tensor theory of the form

2.23 $$\begin{aligned}&\displaystyle S=\int \, \mathrm{d}^4x\sqrt{-g}\left[ \frac{R}{16\pi G}-\frac{1}{2}\partial _\mu \phi \partial ^\mu \phi -V(\phi )\right] +S{_\mathrm{m}}[e^{\sqrt{\frac{2}{3}}\frac{\phi }{{M_\mathrm{{pl}}}}}]\quad \text {with}\qquad \qquad \end{aligned}$$

2.24 $$\begin{aligned}&\displaystyle V(\phi )=\frac{{M_\mathrm{{pl}}}^2}{2}\frac{\phi f'(\phi )-f(\phi )}{(1+f'(\phi ))^2}, \end{aligned}$$

where the canonically-normalised field

2.25 $$\begin{aligned} \phi =-\sqrt{\frac{3}{2}}{M_\mathrm{{pl}}}\ln \left( 1+f'(\varphi )\right) . \end{aligned}$$

The theory is then a chameleon with $M_c = \sqrt{6}{M_\mathrm{{pl}}}$. The Hu–Sawicki model corresponds to a chameleon with $n=-b/(1+b)$ so that only a narrow range in the chameleon theory space is covered, i.e, $-1<n<-1/2$. The most well-studied models are $b=1$ ($n=-1/2$) and $b=3$ ($n=-3/4$), although, typically, results are only quoted for $n=1$, and so we will only focus on this model here.

#### UV properties

Screening relies on the presence of non-linear self-interactions of the scalar field, and on coupling the scalar to the matter energy momentum tensor. Written in the Einstein frame, this necessarily introduces non-renormalisible operators, meaning that additional physics is required in order to UV complete the model (Joyce et al. 2015). Additionally, we might worry that integrating out physics in the UV changes the form of the low-energy theory, either rescaling the coefficients, or introducing new terms into the Lagrangian.

For the theory to be fully predictive, it is important to understand whether the low-energy theory we study is protected from corrections coming from UV physics. One commonly used way to estimate the size of these effects is to compute the Coleman–Weinberg (Coleman and Weinberg 1973) corrections to the scalar mass (Upadhye et al. 2012a). To do this, one computes the corrections to the scalar mass from scalar fields running in loops, these loop corrections arise precisely because the scalar field has non-trivial self interactions in its potential. The Coleman–Weinberg corrections are found to be at least logarithmically divergent with scale. Even if these corrections to the mass are assumed to be small at some scale, they may become important at another scale, or in another environment.

In Upadhye et al. (2012a), the relevance of these corrections for the Eöt-Wash experiment was computed. With some simple assumptions about the scale at which the logarithmic terms become important, it was shown that the current constraints from these experiments are computed in a regime in which the quantum corrections are indeed under control. However, as the experimental sensitivity improves these corrections will become more relevant.

Keeping track of the quantum corrections is also important in order to understand the behaviour of the chameleon in the early universe. In Erickcek et al. (2014, 2013), it was shown that, with the exception of gravitationally coupled chameleons, it is not possible to evolve the chameleon through the radiation dominated era without knowing the UV completion of the model. This is because the decoupling of standard model particles during this epoch give a large impulse to the otherwise slowly rolling chameleon field (Brax et al. 2004b). This causes the chameleon scalar to rapidly roll to the part of the potential where the field’s self interactions are large, and so high energy quantum fluctuations of the field are excited. It is possible that some non-perturbative physics could resolve this, but in the absence of a proof of this, we do not know how to evolve the chameleon model from the early universe to late times in a predictive way. One model, which can evade this problem, is the case $n=-4$ due to the absence of a low-mass scale (that is problematic in the early Universe when energies are typically high) (Miller and Erickcek 2016).

The most reliable way to compute UV corrections to the low-energy chameleon model would be to know exactly what the UV-completion of the theory is. A number of attempts have been made to embed the chameleon mechanism within string theory (Brax et al. 2004a; Conlon and Pedro 2011; Hinterbichler et al. 2011b; Nastase and Weltman 2015, 2013), within supersymmetry (Brax et al. 2013b, a), and using non-canonical kinetic terms (Padilla et al. 2016), but, as yet, no complete theory exists.

### Symmetron screening

The symmetron model does not rely on varying mass, instead, the screening works by suppressing the coupling to matter in high-density regions. This is accomplished using ${\mathbb {Z}}_2$ symmetry restoration. The bare potential and coupling function are

2.26 $$\begin{aligned} V(\phi )=-\frac{1}{2}\mu ^2\phi ^2+\frac{\lambda }{4}\phi ^4;\quad A(\phi )=1+\frac{\phi ^2}{2M_\mathrm{s}^2} \end{aligned}$$

so that the effective potential is

2.27 $$\begin{aligned} V_\mathrm{eff}(\phi )=\frac{1}{2}\mu ^2\left( 1-\frac{\rho }{\mu ^2M_\mathrm{s}^2}\right) \phi ^2+\frac{\lambda }{4}\phi ^4. \end{aligned}$$

This is ${\mathbb {Z}}_2$ ($\phi \rightarrow -\phi$) symmetric (as are $V(\phi )$ and $A(\phi )$ individually). The coefficient of the quadratic term can be either positive or negative depending on the density and, in particular, there is a critical density

**Fig. 2:**
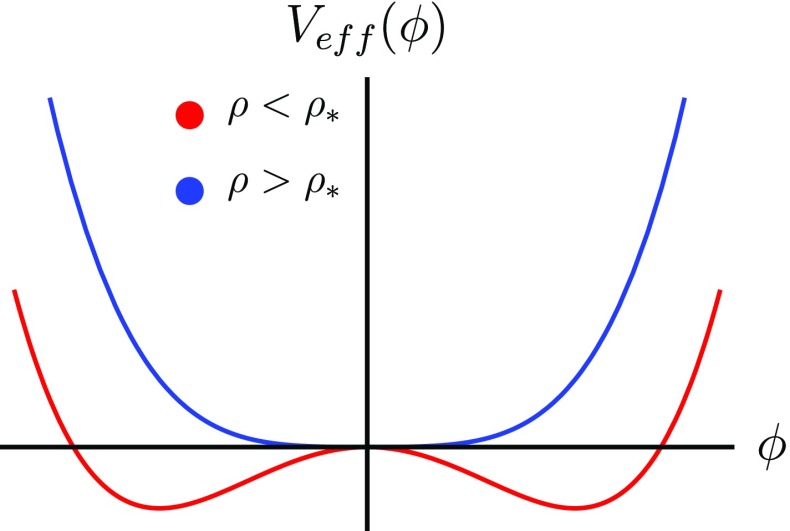
The effective potential for the symmetron when $\rho <\rho _\star$ (red, lower) and when $\rho >\rho _\star$ (blue, upper)

2.28 $$\begin{aligned} \rho _\star \equiv \mu ^2 M_\mathrm{s}^2, \end{aligned}$$

where the sign changes. The screening mechanism is best exemplified by examining the shape of the effective potential sketched in Fig. 2. When $\rho <\rho _\star$ there are two degenerate minima at

2.29 $$\begin{aligned} \phi _\mathrm{min}^{\pm }= & {} \pm \frac{\mu }{\sqrt{\lambda }}\sqrt{1-\frac{\rho }{\mu ^2M_\mathrm{s}^2}} \end{aligned}$$

2.30 $$\begin{aligned}\approx & {} \pm \frac{\mu }{\sqrt{\lambda }}, \;\;\;\;\; \text{ if } \rho \ll \rho _\star \end{aligned}$$

In this case, the ${\mathbb {Z}}_2$ symmetry is spontaneously broken and the coupling to matter is

2.31 $$\begin{aligned} \beta (\phi _0)=\left| \frac{{M_\mathrm{{pl}}}\phi _\mathrm{min}^\pm }{M_\mathrm{s}^2}\right| \approx \frac{\mu {M_\mathrm{{pl}}}}{\lambda M_\mathrm{s}^2}, \end{aligned}$$

giving rise to a fifth-force potentially stronger than gravity. When $\rho >\rho _\star$ the only minimum is at $\phi =0$ so that the symmetry is restored and the coupling $\beta (\phi _0)=0$. In which case no fifth force can be sourced. One can tune the parameters $\mu$, and $\lambda$ in terms of $M_\mathrm{s}$ to ensure that $\rho _\star$ coincides with the present day cosmological density, or so that the fifth-force is of gravitational strength (Hinterbichler and Khoury 2010; Hinterbichler et al. 2011a), but we shall not do so here since we are considering a range of different experimental tests that constrain the parameters in very different environments and on many different scales.

#### Generalized symmetrons

The symmetron screening mechanism is by no means reliant on the specific form of the effective potential (2.27). Indeed, clearly any effective potential of the form

2.32 $$\begin{aligned} V_\mathrm{eff}(\phi )= -\mu ^4\left( 1-\frac{\rho }{\mu ^{4-2n}M_\mathrm{s}^{2n}}\right) \frac{\phi ^{2n}}{\mu ^{2n}}+\frac{\phi ^{2m}}{\varLambda _\mathrm{s}^{2m-4}}, \end{aligned}$$

with $n<m$ and $n,\, m\in {\mathbb {Z}}^+$, exhibits qualitatively similar features to the canonical symmetron. Such an effective potential can arise through the bare potential and coupling functions

2.33 $$\begin{aligned} V(\phi )=-\frac{\phi ^{2n}}{\mu ^{2n-4}}+\frac{\phi ^{2m}}{\varLambda _\mathrm{s}^{2m-4}}; \;\;\;\;\;A(\phi ) = 1 + \frac{\phi ^{2n}}{M_\mathrm{s}^{2n}}. \end{aligned}$$

First discovered by Brax et al. (2012a, b) using tomographic methods, there has been little investigation of these models at the present time and so we do not consider them further here.

#### Radiatively-stable symmetrons

The symmetron model, as described here, suffers the same UV stability properties as the chameleon. In particular, that Coleman–Weinberg corrections could dramatically alter the shape of the potential needed for the symmetron mechanism to work. In this case, however, the one-loop corrections can also be exploited to give rise to the screening in a radiatively stable way (Burrage et al. 2016a).

The Coleman–Weinberg model (Coleman and Weinberg 1973) was originally discussed as a way of using radiative corrections to generate a spontaneous symmetry breaking transition. The classical model is scale invariant, but the one-loop corrections generate a scale through dimensional transmutation of the logarithmic divergences. In the one-field model, higher-order loop corrections become important in the spontaneously broken vacuum, but in a multi-field model these can be kept under control (Garbrecht and Millington 2015), and the one-loop potential can undergo a symmetry breaking transition whilst the higher-order loop corrections remain small.

The radiatively stable symmetron has a different bare potential to that discussed above

2.34 $$\begin{aligned} V(\phi ) = \left( \frac{\lambda }{16 \pi }\right) ^2 \phi ^4\left( \ln \frac{\phi ^2}{m^2}-\frac{17}{6}\right) \end{aligned}$$

however, overall the phenomenology this gives rise to is very similar to that of the standard symmetron.

### Coupling to photons

A conformally coupled scalar field does not have a classical coupling to photons. This is because the scalar couples to the trace of the energy momentum tensor of the matter fields, and photons, being relativistic, have a traceless energy momentum tensor. This is not the end of the story, however, as quantum effects make it easy to generate such a coupling. One way to do this, is to assume the presence of a new heavy fermion which has an electromagnetic charge. Then, an interaction between one conformally coupled scalar, and two photons can be mediated by a triangle-loop of the heavy fermion. If the fermion is sufficiently heavy that it can be integrated out, to leave the Standard Model plus the chameleon as a low-energy effective theory, then the low-energy theory has a contact interaction between the chameleon and two photons (Brax et al. 2010c). Such heavy, charged fermions are ubiquitous in theories of physics beyond that Standard Model, including, string theory, supersymmetry and GUTs. It can also be shown that the Weyl rescaling that allows us to change from Jordan to Einstein frame, gives rise to a coupling to photons after quantisation of these fields, this was shown for the chameleon in Brax et al. (2011b), following earlier work by Kaplunovsky and Louis (1994) in the context of supersymmetry.

The coupling to photons, which arises in all of these cases, is that of a scalar axion-like particle

2.35 $$\begin{aligned} {\mathcal {L}}\supset \frac{\phi }{M_{\gamma }} F_{\mu \nu }F^{\mu \nu } \;. \end{aligned}$$

(For a symmetron model with ${\mathbb {Z}}_2$ symmetry, the leading coupling would instead be quadratic in $\phi$.) Here, $M_{\gamma }$ is the energy scale that controls the coupling to photons, this is not necessarily the scale at which the chameleon couples to other matter particles $M_\mathrm{c}$. The coupling in Eq. (2.35) means that existing constraints on axion-like particles can be applied to the chameleon, although some care must be taken in doing this as standard axion-like particles have fixed mass and couplings, and so constraints from environments of vastly different density can be easily combined. This is not the case for a screened scalar. This axion-like coupling is not necessary for a screening mechanism to work, however, it is difficult to forbid such a coupling in a truly quantum theory. Including the coupling opens new avenues for detecting the chameleon, as high precision searches for axions and axion-like particles can be exploited to detect or constrain the chameleon. For example, the interaction in Eq. (2.35) means that chameleons can be produced through the Primakov effect as photons propagate through a magnetic field. This underlies a range of different experimental search strategies.

## Screening

In this section, we discuss screening mechanisms in the context of astrophysical objects and typical laboratory configurations, and discuss some salient features that are specific to screening mechanisms.

### Astrophysical screening: the thin-shell effect

Astrophysical objects are typically spherical and so, in this section, we consider the screening of a non-relativistic, static, spherically symmetric object of mass ${\mathcal {M}}$, radius *R*, and density $\delta \rho (r)$ immersed in a much larger medium with density $\bar{\rho }$. The total density is $\rho =\bar{\rho }+\delta \rho$. This could represent a star inside a galaxy or a galaxy/dark matter halo/cluster embedded in the cosmological background, in which case $\bar{\rho }$ is the mean cosmic density. We follow the method of Hui et al. (2009), Davis et al. (2012), Sakstein (2013, 2014a), and Burrage and Sakstein (2016). (Other derivations using slightly different procedures recover the same results (Brax et al. 2012b), but the current astrophysical constraints have been derived using the method we present here.) Far away from the object, the field minimizes the effective potential so that one has $\phi (r)\rightarrow \bar{\phi }\equiv \phi _\mathrm{min}(\bar{\rho })$. Near the object, the equation of motion in Eq. (2.6) becomes (in spherical coordinates)

3.1 $$\begin{aligned} \nabla ^2\phi =\frac{1}{r^2}\frac{\, \mathrm{d}}{\, \mathrm{d}r}\left( r^2\frac{\, \mathrm{d}\phi }{\, \mathrm{d}r}\right) =\frac{\, \mathrm{d}V}{\, \mathrm{d}\phi } +\frac{\beta (\phi )\rho }{{M_\mathrm{{pl}}}}. \end{aligned}$$

One can then envisage two regimes. If the field can reach the minimum of the effective potential inside the object, then one has $V_\mathrm{eff}'(\phi )=0$ and the right-hand side of (3.1) is unsourced so that $\phi =\phi _\mathrm{min}(\rho )$ is constant and there is no fifth-force. If instead the field remains close to $\bar{\phi }$, we can linearise $\phi =\bar{\phi }+\varphi$ to find

3.2 $$\begin{aligned} \frac{1}{r^2}\frac{\, \mathrm{d}}{\, \mathrm{d}r}\left( r^2\frac{\, \mathrm{d}\phi }{\, \mathrm{d}r}\right) = m_0^2\varphi +\frac{\beta (\phi _0)}{{M_\mathrm{{pl}}}}\delta \rho , \end{aligned}$$

where $m_0^2=V''(\phi )$. $V(\phi )$ is typically chosen so that $\phi$ is cosmologically relevant, i.e., $m_0 R\ll 1$ and one can ignore the first term on the right-hand side of (3.2), in which case one is left with a Poisson equation

3.3 $$\begin{aligned} \frac{1}{r^2}\frac{\, \mathrm{d}}{\, \mathrm{d}r}\left( r^2\frac{\, \mathrm{d}\phi }{\, \mathrm{d}r}\right) = \frac{\beta (\bar{\phi })}{{M_\mathrm{{pl}}}}\delta \rho . \end{aligned}$$

In practice, we expect a hybrid of these two cases where the field sits close to the minimum of the effective potential at the centre of the object and remains there up to some radius $r_\mathrm{s}$ at which it begins to roll towards its asymptotic value and enter the second regime. There is, therefore, no fifth-force interior to $r_\mathrm{s}$; for this reason we will refer to $r_\mathrm{s}$ as the *screening radius*. Outside the screening radius one can integrate (3.3) once to find

3.4 $$\begin{aligned} \frac{\, \mathrm{d}\phi }{\, \mathrm{d}r}=\frac{\beta (\bar{\phi })\left( {\mathcal {M}}(r)-{\mathcal {M}}(r_\mathrm{s})\right) }{4\pi {M_\mathrm{{pl}}}r^2}, \end{aligned}$$

where

3.5 $$\begin{aligned} {\mathcal {M}}(r)=\int _0^r 4\pi r'^2\delta \rho (r')\, \mathrm{d}r';\quad {\mathcal {M}}\equiv M(R). \end{aligned}$$

The fifth-force (2.5) is then

3.6 $$\begin{aligned} F_5=\frac{2\beta ^2(\bar{\phi })G\left[ {\mathcal {M}}(r)-{\mathcal {M}}(r_\mathrm{s})\right] }{r^2}. \end{aligned}$$

The field equation is only sourced by the density outside the screening radius and so only the mass exterior to this contributes to the fifth-force. Objects that have $r_\mathrm{s}\ll R$ have

**Fig. 3:**
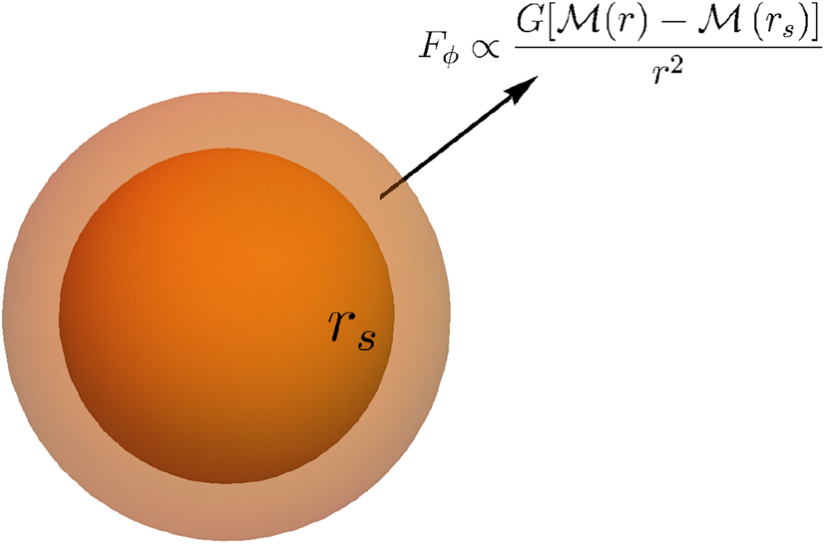
The thin-shell effect. The fifth-force only receives a contribution from the mass in the thin-shell $r_\mathrm{s}<r<R$

3.7 $$\begin{aligned} \frac{F_5}{F_\mathrm{N}}\approx 2\beta ^2(\bar{\phi }) \end{aligned}$$

and are hence unscreened, whereas those for which $r_\mathrm{s}\approx R$ have $F_5/F_N\ll 1$ and are hence screened. In this case, the fifth-force only receives contributions from the mass in a very thin shell outside the screening radius. This phenomena has been dubbed *the thin-shell effect*; we depict this in Fig. 3. Outside the object, the mass term in (3.4) is more important than the density and one has

3.8 $$\begin{aligned} F_5=\frac{2\beta ^2(\bar{\phi })G\left[ {\mathcal {M}}-{\mathcal {M}}(r_\mathrm{s})\right] }{r^2}e^{-m_\mathrm{eff}(r-R)}. \end{aligned}$$

In order to determine whether an object is screened, we must calculate $r_\mathrm{s}$. This can be accomplished by integrating (3.4) from $r_\mathrm{s}$ (where $\phi =\phi _\mathrm{s}\approx \phi _\mathrm{min}(\rho )$) to $\infty$ to find

3.9 $$\begin{aligned} \bar{\phi }-\phi _\mathrm{s}=\frac{\beta (\bar{\phi }){\mathcal {M}}(r_\mathrm{s})}{4\pi {M_\mathrm{{pl}}}r_\mathrm{s}}+\int _{r_\mathrm{s}}^\infty \frac{\beta (\bar{\phi }){\mathcal {M}}(r')}{4\pi r'^2}\, \mathrm{d}r'. \end{aligned}$$

Performing integration by parts and using Eq. (3.5) one finds an implicit relation for $r_\mathrm{s}$

3.10 $$\begin{aligned} \chi \equiv \frac{\bar{\phi }}{2\beta (\bar{\phi }){M_\mathrm{{pl}}}}=4\pi G\int _{r_\mathrm{s}}^\infty r'\delta \rho (r')\, \mathrm{d}r', \end{aligned}$$

where we have ignored $\phi _\mathrm{s}$, since the screening mechanisms always act to push $\phi$ to smaller values inside dense objects. Alternatively, one can use the relation $\, \mathrm{d}\varPhi _\mathrm{N}/\, \mathrm{d}r= G{\mathcal {M}}(r)/r$ to write (3.9) as

3.11 $$\begin{aligned} \chi =-\varPhi _\mathrm{N}(r_\mathrm{s})-r_\mathrm{s}\varPhi _\mathrm{N}'(r_\mathrm{s}). \end{aligned}$$

If (3.11) [or, equivalently, (3.10)] has no solution then $r_\mathrm{s}=0$ and the object is fully unscreened. Given that $\varPhi _\mathrm{N}<0$ whilst $\varPhi _\mathrm{N}'>0$ there can be no solution when $\chi >\varPhi _\mathrm{N}=G{\mathcal {M}}/R$. Hence, only objects where $\chi <G{\mathcal {M}}/R$ can be partially (or fully for $\chi \ll G{\mathcal {M}}/R$) screened.

The screening criteria above is particularly useful for determining which astrophysical objects will be partially unscreened and for which range of parameters; one simply needs to calculate the Newtonian potential. Commonly studied examples are given in Table 1. In the case of main sequence stars, one can find the Newtonian potential using the mass-radius relation

3.12 $$\begin{aligned} \frac{M}{M_\odot }=\left( \frac{R}{R_\odot }\right) ^{\nu }, \end{aligned}$$

where $\nu$ depends on the type of star in question. In the case of galaxies, one can use the Virial theorem to calculate the Newtonian potential from the circular velocity:

3.13 $$\begin{aligned} v^2=\frac{G{\mathcal {M}}}{R}. \end{aligned}$$

Dwarf galaxies are particularly good probes due to their low Newtonian potentials. Indeed, many of the astrophysical tests we will discuss below use either dwarf galaxies themselves or their constituent objects. Setting $\bar{\phi }=\phi _0$ the parameter of interest is

3.14 $$\begin{aligned} \chi _0\equiv \frac{\phi _0}{2\beta (\phi _0){M_\mathrm{{pl}}}}. \end{aligned}$$

Unscreened dwarf galaxies can then, in theory, test .

**Table 1:** Astrophysical objects of interest and their Newtonian potentials

Object	$\varPhi _\mathrm{N}$
Earth	$10^{-9}$
Moon	$10^{-11}$
Main-sequence stars ($M_\odot$)	$10^{-6}$
post Main-sequence stars (1–$10 M_\odot$)	$10^{-7}$–$10^{-8}$
Spiral and elliptical galaxies	$10^{-6}$
Dwarf galaxies	$10^{-8}$

In practice, one also needs to worry about environmental screening. So far, we have only considered the screening of a single object embedded in a larger background, but real astrophysical objects are typically not isolated; galaxies are found in clusters and stars come in pairs or groups. The non-linear nature of the field equations means that we cannot simply superimpose solutions sourced by different objects to obtain a new solution. This implies that an object’s environment can affect whether it is screened or not. The most important example of this is the screening of dwarf galaxies. Taken in isolation, the Newtonian potential for a dwarf galaxy is $\mathcal {O}(10^{-8})$ but the typical potential associated with clusters of galaxies is $\mathcal {O}(10^{-4})$ so that only values of $\chi _0$ larger than this can be tested. The ideal probes are, therefore, dwarf galaxies located in voids that do not suffer from environmental screening. There has been a great effort towards determining the criteria for environmental screening (Li et al. 2012; Lombriser et al. 2012a, 2013; Cai et al. 2015). Most of these rely on numerical N-body simulations, whose description lies outside the scope of this review, but the end result is a *screening map* (Cabre et al. 2012) of the local universe that classifies galaxies as either screened, partially screened, or unscreened. To date, all astrophysical tests using dwarf galaxies have been taken from this screening map.

Astrophysical tests ultimately end up constraining regions in the $\chi _0$–$\beta (\phi _0)$ plane. For our models of interest, one has

3.15 $$\begin{aligned} \beta (\phi _0)={\left\{ \begin{array}{ll} \frac{{M_\mathrm{{pl}}}}{M_\mathrm{c}} &{} \quad \text {Chameleons }\\ \frac{\mu {M_\mathrm{{pl}}}}{\sqrt{\lambda }M_\mathrm{s}^2} &{} \quad \text {Symmetrons}\\ \end{array}\right. }, \end{aligned}$$

and

3.16 $$\begin{aligned} \chi _0={\left\{ \begin{array}{ll} \frac{1}{2}\left( \frac{M_\mathrm{c}}{{M_\mathrm{{pl}}}^2}\right) ^{\frac{n+2}{n+1}}\left( \frac{n\varLambda ^{n+4}}{3\varOmega {_\mathrm{m}}H_0^2}\right) ^{\frac{1}{n+1}} &{} \quad \text {Chameleons }\\ \frac{1}{2}\left( \frac{M_\mathrm{s}}{{M_\mathrm{{pl}}}}\right) ^{2} &{} \quad \text {Symmetrons}\\ \end{array}\right. }, \end{aligned}$$

where we have replaced the cosmic density in $\phi _\mathrm{min}(\rho )$ with $3\varOmega {_\mathrm{m}}{M_\mathrm{{pl}}}^2H_0^2$.

#### Screening in *f*(*R*) theories

Given that *f*(*R*) models only cover a restricted range of *n* and have a fixed value of $M_c$, it is not particularly enlightening to constrain *f*(*R*) theories in terms of $\varLambda$ and *n*, even more so, since the cosmological constant is fixed by tuning the parameters so that $\varLambda =2.4\times 10^{-3}$ eV does not have any special significance. (In this sense, *f*(*R*) theories should be thought of as describing deviations from the $\varLambda$CDM model). Instead, constraints are often quoted in terms of the parameter $f_{R0}=f'(R_0)$, the first derivative of *f*(*R*) evaluated at the present time in the cosmological background. The significance of this parameter can be seen by examining the screening in the *f*(*R*) formalism. Consider an object of density $\rho _0$ embedded in the cosmological background where the Ricci scalar curvature is $R_0$ and the density is $\rho _0$. If one embeds an object with density $\delta \rho$ into this background, then it will source a Newtonian potential ($g_{00}=-a^2(1+2\varPhi )$) and perturb $R=R_0+\delta R$, $f_R=f_{R0}+\delta f_R$ (Schmidt 2010) such that

3.17 $$\begin{aligned} \nabla ^2\varPhi&=\frac{16\pi G}{3}\rho -\frac{1}{6}\delta R(f_{R0}) \end{aligned}$$

3.18 $$\begin{aligned} \nabla ^2 \delta f_R&=\frac{1}{3}\left( \delta R(f_{R0})-8\pi G\delta \rho \right) . \end{aligned}$$

In the limit where $\delta f_R\ll f_{R0}$, there can be no source for $\delta f_R$ and one has $\delta R(f_{R0})=8\pi G\delta \rho$ so that (3.17) becomes $\nabla ^2\varPhi =4\pi G\delta \rho$. Therefore, in this limit we recover the Poisson equation and there are no deviations from GR; the fifth force is screened. In the opposite limit where $\delta f_R\gg f_{R0}$, we can expand $\delta R (f_R)\approx \delta f_R/f_{RR}$ so that Eq. (3.18) becomes

3.19 $$\begin{aligned} \nabla ^2 \delta f_R&=m_f^2\delta f_R-\frac{8\pi G}{3}\delta \rho ,\quad m_f^2=\frac{1}{3 f_{RR}}, \end{aligned}$$

which is the equation of motion for a massive scalar with mass $m_f$. On scales shorter than $m_f^{-1}$ the mass can be ignored and one finds, using (3.17), $\nabla ^2\varPhi =16\pi G\delta \rho /3$ so that the Newtonian potential is enhanced by a factor of 4 / 3; the force is fully unscreened. Note that, in this limit, Eq. (3.18) gives $|\delta f_R|=2\varPhi /3$ but the maximal value of $\delta f_R$ is $f_{R0}$ so we conclude that objects must be partially screened if $f_{R0}<2\varPhi /3$. Thus, we see that $f_{R0}$ is the *f*(*R*) equivalent of the $\chi$.

#### Gravitational lensing: dynamical versus lensing masses

Conformal transformations leave null geodesics unchanged (Padmanabhan 2010) ($\tilde{g}_{\mu \nu }\dot{x}^\mu \dot{x}^\nu =A^2(\phi )g_{\mu \nu }\dot{x}^\mu \dot{x}^\nu =0$) so that photons move on geodesics of both $\tilde{g}_{\mu \nu }$ and $g_{\mu \nu }$. This has some novel implications for gravitational lensing by massive bodies. Expanding the Einstein frame metric in the Newtonian gauge:

3.20 $$\begin{aligned} \, \mathrm{d}s^2=(-1+2\varPhi _\mathrm{N})\, \mathrm{d}t^2 + (1+2\varPsi _\mathrm{N})\, \mathrm{d}x^2, \end{aligned}$$

the Jordan frame metric is

3.21 $$\begin{aligned} \, \mathrm{d}\tilde{s}^2=\left( -1+2\varPhi _\mathrm{N}-2\beta (\bar{\phi })\frac{\phi }{{M_\mathrm{{pl}}}}\right) \, \mathrm{d}t^2 + \left( 1+2\varPsi _\mathrm{N}+2\beta (\bar{\phi })\frac{\phi }{{M_\mathrm{{pl}}}}\right) \, \mathrm{d}x^2,\nonumber \\ \end{aligned}$$

where we have set $\phi \rightarrow \bar{\phi }+\phi$ and have absorbed factors of $A(\phi _0)^2$ into *t* and $x^i$ (see Sect. 3.2). We can, thus, identify the Jordan frame potentials

3.22 $$\begin{aligned} \tilde{\varPhi }_\mathrm{N}&=\varPhi _\mathrm{N}-\beta (\bar{\phi })\frac{\phi }{{M_\mathrm{{pl}}}}\quad \tilde{\varPsi }=\varPsi +\beta (\bar{\phi })\frac{\phi }{{M_\mathrm{{pl}}}}. \end{aligned}$$

The Newtonian potential, which governs the motion of non-relativistic particles, therefore, depends on $\phi$ whereas the lensing potential, $\varPsi$, which governs the motion of photons is

3.23 $$\begin{aligned} \tilde{\varPsi }_\mathrm{L}=\frac{1}{2}\left( \tilde{\varPhi }_\mathrm{N}+\tilde{\varPsi }\right) =\varPhi _\mathrm{N}, \end{aligned}$$

where we have used the relationship $\varPsi _\mathrm{N}=\varPhi _\mathrm{N}$, which is a result of working in the Einstein frame. For an extended object of mass ${\mathcal {M}}$, the mass inferred from lensing is the true mass ${\mathcal {M}}$ because the Einstein frame potentials satisfy the Poisson equation. Conversely, the potential governing the motion of non-relativistic objects satisfies

3.24 $$\begin{aligned} \tilde{\varPhi }_\mathrm{N}'=\frac{G{\mathcal {M}}}{r^2}A^2(\bar{\phi })\left[ 1+2\beta ^2(\bar{\phi })\left( 1-\frac{{\mathcal {M}}(r_\mathrm{s})}{{\mathcal {M}}}\right) \right] =\frac{G{\mathcal {M}}_\mathrm{dyn}}{r^2}, \end{aligned}$$

which defines a dynamical mass ${\mathcal {M}}_\mathrm{dyn}\ge {\mathcal {M}}$ with equality for fully screened objects. The difference between the lensing and dynamical masses is in stark contrast to GR, and is a particularly useful feature for testing modified gravity using astrophysical observations.

### Solar-system tests

Classical tests of GR use the PPN formalism applied to solar-system objects and so, in this section, we will illustrate how these tests apply to screened modified gravity, and why they yield only weak constraints.

#### PPN parameters

The PPN metric is both an ansatz (for the possible potentials that could appear in the metric sourced by a massive body) and a gauge choice. There are 10 parameters that can be calculated and compared with observations, but only two are relevant for conformal scalar–tensor theories [disformal theories involve four parameters (Ip et al. 2015]. The PPN metric with these two parameters is (for a spherically symmetric object of mass ${\mathcal {M}}$)

3.25 $$\begin{aligned} g_{00}=-1+2\frac{G {\mathcal {M}}}{r} -2\beta \left( \frac{G{\mathcal {M}}}{r}\right) ^2, \quad g_{0i}=0,\quad \text {and} \quad g_{ij}=\left( 1+2\gamma \frac{G{\mathcal {M}}}{r}\right) \delta _{ij}.\nonumber \\ \end{aligned}$$

The parameter $\gamma$ ($=1$ in GR) sets the amount of light-bending by massive objects, and the Shapiro time-delay effect; and the parameter $\beta$ ($=1$ in GR) measures the amount of non-linearity in the field equations. The term proportional to $\beta$ is responsible for the precession of the perihelion of Mercury. Note that the first term in $g_{00}$ is not free to vary, this is a gauge choice that implies that *G* is Newton’s constant as measured in Cavendish-type experiments.

General expressions for $\gamma$ and $\beta$ in screened scalar–tensor theories can be found in Hees and Fuzfa (2012) and Zhang et al. (2016). It is more instructive, however, to consider the solution for the fifth-force profile of a static object derived in (3.6). We will ignore the mass of the scalar for simplicity but including it does not change any of what follows. The calculation of the fifth-force was performed in the Einstein frame but the PPN metric is defined in the Jordan frame, since it is the metric that controls the geodesics of matter and so our task is to calculate the Jordan frame metric given $\phi$ to $\mathcal {O}(v^2/c^2)$ to find $\gamma$. The calculation of $\beta$ is analogous except one continues to $\mathcal {O}(v^4/c^4)$; this calculation is long and tedious, and one does not gain any additional insight. For this reason, we will only calculate $\gamma$.

To begin, we summarize our Einstein frame solution. This is

3.26 $$\begin{aligned} g_{00}=&-1+2\frac{G{\mathcal {M}}}{r} ,\quad g_{0i}=0,\quad g_{ij}=\left( 1+2\frac{G{\mathcal {M}}}{r}\right) \delta _{ij},\quad \text {and}\nonumber \\ \quad \phi =&\bar{\phi }-\beta (\bar{\phi })\frac{{\mathcal {M}}-{\mathcal {M}}(r_\mathrm{s})}{4\pi {M_\mathrm{{pl}}}r}, \end{aligned}$$

where we have used the fact that $F_5=2\beta (\bar{\phi })\phi '$ to find the field profile. Next, we can expand the metric as

3.27 $$\begin{aligned} \tilde{g}_{\mu \nu }=A^2(\phi )g_{\mu \nu }\approx A^2(\bar{\phi })(1+2\beta (\bar{\phi })\varphi ) g_{\mu \nu }, \end{aligned}$$

where $\varphi =\phi -\bar{\phi }$. The factor of $A^2(\bar{\phi })$ is usually ignored claiming “$A(\bar{\phi })\approx 1$,” but a more correct treatment is to rescale the coordinates such that $t\rightarrow t/A(\bar{\phi })$ and $r\rightarrow r/A(\bar{\phi })$. We also need to rescale the mass ${\mathcal {M}}$, since this was defined using Einstein frame coordinates, and Einstein frame densities. Note that one has $\tilde{T}^{{\mu \nu }}=A^6T^{\mu \nu }$, which implies $\tilde{\rho }=\tilde{g}_{\mu \nu }\tilde{T}^{\mu \nu }=A^4\rho$. The mass then needs to be rescaled as ${\mathcal {M}}\rightarrow A(\bar{\phi }){\mathcal {M}}$. Rescaling the mass and the coordinates, the Jordan frame metric is

3.28 $$\begin{aligned} \tilde{g}_{00}&=-1+2\frac{A^2(\bar{\phi })G{\mathcal {M}}}{r}\left( 1+2\beta (\bar{\phi })^2\left[ 1-\frac{{\mathcal {M}}(r_\mathrm{s})}{{\mathcal {M}}}\right] \right) ,\quad \tilde{g}_{0i}=0,\quad \text {and}\end{aligned}$$

3.29 $$\begin{aligned} g_{ij}&=\left[ 1+2\frac{A^2(\bar{\phi })G{\mathcal {M}}}{r}\left( 1-2\beta (\bar{\phi })^2\left[ 1-\frac{{\mathcal {M}}(r_\mathrm{s})}{{\mathcal {M}}}\right] \right) \right] \delta _{ij}, \end{aligned}$$

where the weak-field limit implies we ignore all higher-order polynomials involving $\phi$. More correctly, the PPN counting scheme assumes $\phi \le GM/r\sim v^2/c^2$ and higher-power terms, and cross terms are, therefore, higher-order.

The Jordan frame metric is not yet in the PPN gauge; we need to rescale

3.30 $$\begin{aligned} G\rightarrow G_\mathrm{N}\equiv A^2(\bar{\phi })\left( 1+2\beta ^2(\bar{\phi })\left[ 1-\frac{{\mathcal {M}}(r_\mathrm{s})}{{\mathcal {M}}}\right] \right) . \end{aligned}$$

This defines Newton’s constant as measured in laboratory experiments. The distinction between *G* and $G_\mathrm{N}$ is not overly important for screened modified gravity because these experiments are performed deep in the screened regime and $G\approx G_\mathrm{N}$ but is crucial for theories without screening mechanisms. Performing this rescaling, one finds a metric in precisely the PPN form with (Saaidi et al. 2011; Hees and Fuzfa 2012; Schärer et al. 2014; Zhang et al. 2016; Sakstein 2017)

3.31 $$\begin{aligned} \gamma&=\left[ 1-2\beta (\bar{\phi })^2\left( 1-\frac{{\mathcal {M}}(r_\mathrm{s})}{{\mathcal {M}}}\right) \right] \left[ 1+2\beta (\bar{\phi })^2\left( 1-\frac{{\mathcal {M}}(r_\mathrm{s})}{{\mathcal {M}}}\right) \right] ^{-1} \nonumber \\&\approx 1-4\beta (\bar{\phi })^2\left( 1-\frac{{\mathcal {M}}(r_\mathrm{s})}{{\mathcal {M}}}\right) . \end{aligned}$$

Note that throughout this derivation we have not made use of any screening mechanisms directly, we could have taken any conformal field theory and applied the same procedure. The novel aspect of screening mechanisms is the non-linearity in the field equations, which means that instead of having $|\gamma -1|\propto 2\beta ^2(\bar{\phi })$, one instead has $|\gamma -1|\propto 2\beta ^2(\bar{\phi })(1-{\mathcal {M}}(r_\mathrm{s})/{\mathcal {M}})\ll 2\beta ^2(\bar{\phi })$ in the screened regime. Without screening mechanisms, we would have to tune $\beta ^2(\bar{\phi })<10^{-5}$ in order to satisfy the Cassini bound $|\gamma -1|<(2.1\pm 2.3)\times 10^{-5}$ (Fomalont et al. 2009). With screening mechanisms, this bound can be automatically satisfied for screened objects (${\mathcal {M}}(r_\mathrm{s})\approx {\mathcal {M}}(r)$) without the need to perform any tunings.

#### Lensing revisited

The careful reader will now be puzzled by a conundrum. We have already argued in Sect. 3.1.2 that screened modified gravity (in fact, our derivation above applies equally to all conformal scalar–tensor theories) does not affect the lensing of light. We have also argued in this section that the PPN parameter $\gamma \ne 1$ so that light bending by the Sun is different than in GR, which implies that the scalar does affect lensing. In fact, both of these statements are compatible, the difference is merely a choice of coordinates.

In Sect. 3.1.2, we did not fix to the PPN gauge, and so what we called *G* is not the same as $G_\mathrm{N}$, the value measured in laboratory experiments (although these should be approximately the same, since we live in a screened environment). In fact, we could equivalently write Eq. (3.24) as

3.32 $$\begin{aligned} \tilde{\varPhi }_\mathrm{N}'=\frac{G_\mathrm{N}{\mathcal {M}}}{r^2}. \end{aligned}$$

This relation is typically tested using kinematics, i.e., by equating it to $v_c^2/r$, where $v_c$ is the circular velocity. Such a test does not determine the mass, but rather, the product $G_\mathrm{N}{\mathcal {M}}=G{\mathcal {M}}_\mathrm{dyn}$. If one chooses to set $G=G_\mathrm{N}$, then this measurement determines ${\mathcal {M}}_\mathrm{dyn}$, and one finds that this is larger than ${\mathcal {M}}$. Alternatively, one could remove *G* completely by measuring $\tilde{\varPsi }=G{\mathcal {M}}_\mathrm{lens}/r$ and take the ratio $\tilde{\psi }/\tilde{\varPhi }_\mathrm{N}={\mathcal {M}}_\mathrm{lens}/{\mathcal {M}}_\mathrm{dyn}=\gamma$. Only the ratio of the two metric potentials is relevant physically, that is to say, the amount of gravitational lensing relevant to the force felt by non-relativistic objects. Whether or not $\phi$ directly affects lensing or not is completely a matter of coordinates, and how one chooses to interpret them.

### Equivalence principle violations

One important feature of screened modified gravity models is that they do not satisfy the equivalence principle. By this, we mean that extended objects with identical masses but differing compositions will not fall at the same rate in externally applied gravitational (Newtonian + scalar) fields.^5^ This can be quantified by considering the Newtonian equation of motion for an extended object in external fields $\varPhi _\mathrm{N}^\mathrm{ext}$ and $\phi ^\mathrm{ext}$ (defined in the Einstein frame) respectively

3.33 $$\begin{aligned} {\mathcal {M}}\ddot{\mathbf {r}}=-{\mathcal {M}}\nabla \varPhi _\mathrm{N}^\mathrm{ext}-Q\nabla \phi ^\mathrm{ext}. \end{aligned}$$

The mass on the left-hand side is the inertial mass of the object, whereas the mass on the right-hand side is the gravitational mass, which can be thought of as a gravitational charge (analogous to the electric charge) for the object. Since we are working in the Einstein frame, these two are equal. The quantity *Q* is the object’s scalar charge, which describes its response to the externally applied scalar gradient; one can show that (Hui et al. 2009)

3.34 $$\begin{aligned} Q=\beta (\phi _0)\left( {\mathcal {M}}-{\mathcal {M}}(r_\mathrm{s})\right) . \end{aligned}$$

This implies that the motion of the object depends on the screening radius, which in turn depends on the objects internal structure. The equivalence principle is thus violated for all objects except those that are completely screened (because $Q=0$) or fully unscreened (because $r_\mathrm{s}=0$ and $Q={\mathcal {M}}$). This equivalence principle violation allows for several novel tests that we will discuss below.

### Laboratory screening

Laboratory searches for screened fifth forces, and the particles that mediate them, are typically performed in a vacuum chamber. Inside this chamber, the position of the minimum of the effective potential can be different to the minimum of the effective potential in the walls of the vacuum chamber and its environment. This is the key difference between screening in the laboratory, and screening in other astrophysical environments; in a vacuum chamber there is a region of low density surrounded by a region of higher density.

The behaviour of the field in the experimental apparatus depends on its mass, as the corresponding Compton wavelength sets the scale over which the field can vary its value. The field can only change its value from the exterior of the experiment to the interior of the walls of the vacuum chamber if its Compton wavelength in the walls is of order the thickness of the walls or smaller. Similarly, the field can only vary its value from the walls to the vacuum at the center of the chamber if its Compton wavelength in the chamber is comparable to, or smaller, than the diameter of the chamber.

The chameleon field can vary its mass much more easily than the symmetron, and as a result laboratory tests constrain a much broader range of models for the chameleon. If the symmetron mass is too small it will not be able to vary its VEV over the scale of the experiment. In this case, there are no field gradients in the experiment, and no resulting fifth forces, so no constraints can be placed. As the symmetron mass increases the vev starts to vary within the experiment, and a fifth force is present, however this fifth force may then be exponentially suppressed by the Yukawa term $e^{-m r}$, where *m* is the mass of the symmetron in the vacuum. In general, therefore, laboratory experiments will only constrain a small range of symmetron masses (Upadhye 2013; Burrage et al. 2016b; Brax and Davis 2016).

The chameleon field can vary more easily in a laboratory vacuum, and therefore is much more amenable to laboratory constraints. Over a wide range of the chameleon parameter space, the chameleon will not be able to reach the value that minimises its potential in the interior of the vacuum chamber, and instead it will evolve to the value that sets its mass to be of order the size of the chamber. Once the corresponding Compton wavelength becomes smaller than the size of the chamber, the field is able to reach the minimum of its effective potential.

If the experiment is performed in a sufficiently small region at the center of the vacuum chamber, then we can assume that the background value due to the vacuum chamber is constant. Then, the screening condition simplifies. A sphere at the center of the vacuum chamber will be screened if there is a solution for the screening radius $r_\mathrm{s}>0$ to

3.35 $$\begin{aligned} 1-\frac{r_S^2}{R^2}=\left( \frac{M_\mathrm{c}}{M_P}\right) ^2\frac{8\pi M_P^2 R}{M_\mathrm{obj}}\left( \frac{\phi _\mathrm{vac}-\phi _\mathrm{min}(\rho _\mathrm{obj})}{M}\right) , \end{aligned}$$

where $M_\mathrm{obj}$ is the mass of the sphere, *R* its radius and $\rho _\mathrm{obj}$ its density. $\phi _\mathrm{vac}$ is the background chameleon value due to the vacuum chamber. The right-hand side of this can be viewed as the ratio of the chameleon to Newtonian potentials at the surface of the object; this relation can be found by evaluating Eq. (3.10) for a sphere of constant density.

Clearly determining both the background value of the scalar field and the condition for screening becomes more complicated for non-spherical geometries, and in these cases, numerics are needed to place definitive constraints. However, the principles described here will still guide the shape of the field profile and the conditions for screening.

Laboratory searches for fifth forces are performed with both classical and quantum experiments. To determine the condition for screening in a quantum experiment requires a little more thought. If the experiment is sufficiently low energy that the internal structure of the source is not disrupted, it must still be checked how the chameleon screening condition is affected by the delocalisation of the object’s center of mass (Burrage et al. 2015). The chameleon can respond to changes in the position of the source on timescales on the order of $1/ m_\mathrm{eff}(\phi _\mathrm{vac})$, and a delocalised source can be considered to fluctuate around with a time-scale $R_\mathrm{trap}/v$, where $R_\mathrm{trap}$ is the spatial extent of the trapping potential, and *v* is the velocity of the particle. If $(v/R_\mathrm{trap})<m_\mathrm{vac}$, the chameleon field can respond to the quantum fluctuations of the object and, therefore, it is the object’s density and size that determine whether the object is screened, regardless of the uncertainty on its center-of-mass position. Otherwise, the chameleon cannot respond to the fluctuations in the position of the source, and the relevant density in the screening condition is $\bar{\psi }_\mathrm{obj}\psi _\mathrm{obj}$, where $\psi _\mathrm{obj}$ is the wavefunction of the object (Burrage et al. 2015).

### Screening in the Jordan frame

In this review, we will work exclusively in the Einstein frame but, for completeness, and because it has received little attention in the literature, we will discuss how screening works in the Jordan frame. We will follow the notation of Hui et al. (2009), who have provided the most comprehensive treatment to date,^6^ although we will not perform the full Einstein–Infeld–Hoffmann approach for extended objects, instead, we will work with the one-body problem to be consistent with our analyses above. Written in the Jordan frame, the action (2.1) is

3.36 $$\begin{aligned} S=\int \, \mathrm{d}^4 x\sqrt{-\tilde{g}}\left[ \frac{{M_\mathrm{{pl}}}^2 }{2A^2(\phi )}\tilde{R}(\tilde{g})-\frac{k(\phi )}{2}\partial _\mu \phi \partial ^\mu \phi -\frac{V(\phi )}{A^4(\phi )}\right] +S_\mathrm{m}[\tilde{g}_{\mu \nu }],\qquad \end{aligned}$$

where

3.37 $$\begin{aligned} k(\phi )=\frac{1}{A^{2}(\phi )}\left[ 1+6{M_\mathrm{{pl}}}^2\left( \frac{\, \mathrm{d}\ln A}{\, \mathrm{d}\phi }\right) ^2\right] . \end{aligned}$$

In the Jordan frame, the matter is minimally coupled to $\tilde{g}_{\mu \nu }$ but the scalar has a non-canonical kinetic term, is non-minimally coupled to *R*, and the scalar potential is $V_\mathrm{J}(\phi )=V(\phi )/A^4(\phi )$. The scalar equation of motion is

3.38 $$\begin{aligned} k(\phi )\Box \phi +\frac{\, \mathrm{d}k}{\, \mathrm{d}\phi }\partial _\mu \phi \partial ^\mu \phi -\frac{\, \mathrm{d}V_\mathrm{J}}{\, \mathrm{d}\phi }+\frac{1}{2}\frac{\, \mathrm{d}A^{-2}(\phi )}{\, \mathrm{d}\phi }\tilde{R}=0. \end{aligned}$$

Since the Ricci scalar appears in this equation, we also need the Einstein equations, which are

3.39 $$\begin{aligned} G_{\mu \nu }= & {} \frac{A^2(\phi )}{{M_\mathrm{{pl}}}^2}\left[ \tilde{T}_{\mathrm{m}\,\,{\mu \nu }}+k(\phi )\partial _\mu \phi \partial _\nu \phi -g_{\mu \nu }\left( \frac{k}{2}\nabla _\alpha \phi \nabla ^\alpha \phi +\,V_\mathrm{J}(\phi )\right) \right. \nonumber \\&\left. +\left( \nabla _\mu \nabla _\nu -g_{\mu \nu }\Box \right) A^{-2}\right] . \end{aligned}$$

Taking the trace of this, one finds

3.40 $$\begin{aligned} \tilde{R}=-\frac{A^2(\phi )}{{M_\mathrm{{pl}}}^2}\left[ \tilde{T}_\mathrm{m}-k\partial _\alpha \phi \partial ^\alpha \phi +V_\mathrm{J}+3\Box A^{-2}(\phi )\right] , \end{aligned}$$

which can be used to eliminate $\tilde{R}$ in Eq. (3.38). These equations are complicated, but they simplify significantly in the Newtonian (weak-field) limit. As discussed by Will (2004); Ip et al. (2015), the expansion parameter in the Newtonian limit is $v^2/c^2$ (or *GM* / *R*, the Newtonian potential) and one should take $\phi \sim v^2/c^2$ or smaller. In this case, one has

3.41 $$\begin{aligned} A^n(\phi )\approx 1+\frac{n\beta (\phi _0)\phi }{{M_\mathrm{{pl}}}},\,V_J(\phi )\approx V(\phi ),\,\partial _\alpha \phi \partial ^\alpha \phi \sim \mathcal {O}\left( \frac{v^4}{c^4}\right) ,\text { and } \tilde{T}_\mathrm{m}\approx -\tilde{\rho }, \end{aligned}$$

where we have neglected terms at higher-order than $v^2/c^2$ and possible time-derivatives of the asymptotic field. We remind the reader that $\tilde{\rho }\sim v^2/c^2$ is the Jordan frame density. In the weak-field limit, we can therefore ignore all factors of $k(\phi )$, since they multiply terms that are higher-order than $v^2/c^2$.^7^ We may ignore this contribution. With these approximations, one has $\tilde{R}\approx -\tilde{T}_\mathrm{m}/{M_\mathrm{{pl}}}\approx \tilde{\rho }/{M_\mathrm{{pl}}}^2$ so that Eq. (3.38) becomes (sending $\Box \rightarrow \nabla ^2$ as time-derivatives are of order *v* / *c* in the Newtonian limit)

3.42 $$\begin{aligned} \nabla ^2\phi =\frac{\, \mathrm{d}V(\phi )}{\, \mathrm{d}\phi }+\frac{\beta (\phi _0)\tilde{\rho }}{{M_\mathrm{{pl}}}}. \end{aligned}$$

This is none other than Eq. (2.7) (the Einstein frame scalar equation of motion) with the Einstein frame density replaces by the Jordan frame density. In fact, since $\tilde{T}_\mathrm{m}^{\mu \nu }=A^{-6}T^{{\mu \nu }}_\mathrm{m}$ one has $\tilde{T}_\mathrm{m} = A^{-4} T_\mathrm{m}$ so that $\tilde{\rho }=\rho +\mathcal {O}(v^4/c^4)$. The equation of motion for the scalar is therefore identical in both frames in the weak-field limit. Non-relativistic screening, which is all we are concerned with in this review, therefore works identically in both frames.

In order to find the fifth-force, one can perform the Weyl-rescaling $\tilde{g}_{\mu \nu }=A^2(\phi )g_{\mu \nu }$ [taking the weak-field limit (3.41)] on Eq. (3.20) to find

3.43 $$\begin{aligned} \, \mathrm{d}\tilde{s}=\left( -1+2\varPhi +2\frac{\beta (\phi _0)\phi }{{M_\mathrm{{pl}}}}\right) \, \mathrm{d}t^2 +\left( 1+2\varPsi -2\frac{\beta (\phi _0)\phi }{{M_\mathrm{{pl}}}}\right) \delta _{ij}\, \mathrm{d}x^i\, \mathrm{d}x^j\qquad \end{aligned}$$

so that the Jordan frame potentials are

3.44 $$\begin{aligned} \tilde{\varPhi }&=\varPhi +\frac{\beta (\phi _0)\phi }{{M_\mathrm{{pl}}}}\mathrm { and}\end{aligned}$$

3.45 $$\begin{aligned} \tilde{\varPsi }&=\varPsi -\frac{\beta (\phi _0)\phi }{{M_\mathrm{{pl}}}}. \end{aligned}$$

In the weak-field limit, the force is

3.46 $$\begin{aligned} F= -\varvec{\nabla }\tilde{\varPhi }=-\nabla \varPhi -\frac{\beta (\phi _0)}{{M_\mathrm{{pl}}}}\varvec{\nabla }\phi . \end{aligned}$$

The second term is the fifth force, which is identical to the total force calculated in the Einstein frame.

## Experimental tests

In this section, we summarize the present experimental tests of chameleon and symmetron screening, which range from particle-collider and precision-laboratory experiments to astrophysical tests using stars and galaxies.

### Fifth-force searches

Fifth-force searches aim to directly measure the force between two objects and search for deviations from Newton’s law. The experiment is performed inside a vacuum chamber to reduce noise, and the geometry of the experiment is designed to minimize the Newtonian force. Recently, some experiments have been designed specifically for the task of searching for chameleons, either by adapting the geometry to maximize the chameleon force, or by varying the density inside the vacuum chamber. Typically, scales of order $\upmu$m or greater are probed.

#### Torsion balance experiments

Torsion balance experiments typically consist of one mass that acts as a pendulum suspended above a second that sources a gravitational field and acts as an attractor. The two masses are arranged in a manner that cancels the inverse-square contribution to the total force so that the experiment is sensitive to any deviations.

The state-of-the-art in torsion balance tests is the Eöt-Wash experiment (Adelberger et al. 2003; Kapner et al. 2007; Lambrecht et al. 2005), which uses two circular disks as test-masses. The disks have holes bored into them which act as missing masses, giving rise to a net torque due to dipole (and higher-order multipole) moments. The upper disk is rotated at an angular velocity such that the contribution from any inverse-square forces to the torque is zero and, therefore, any residual force is non-Newtonian. The absence of any such forces places strong constraints on non-inverse-square law modifications of gravity. This includes any scalar–tensor theory where the field is massive, including Yukawa interactions, and chameleons.

In order to reduce electromagnetic noise, the pendulum and attractor are coated in gold and a beryllium-copper membrane is placed between them. This poses no additional problems for linear theories such as Yuakawa forces, but does present several technical complications for chameleon theories. The membrane may or may not have a thin shell depending on the parameters under study, and the highly non-linear nature of the field equations make the theoretical modelling of this non-symmetric system difficult. Over time, several works have appeared with the aim of improving the accuracy of the theoretical calculation of the chameleon torque (Brax et al. 2008; Adelberger et al. 2007; Mota and Shaw 2006, 2007; Upadhye 2012b), the most recent being the work of Upadhye (2012a), which uses the so-called *one-dimensional plane-parallel* approximation to include the effects of the missing masses on the chameleon force profile. A similar effort has been undertaken for symmetron models, with the most stringent constraints presented in Upadhye (2013).

#### Casimir-force tests

The Casimir force (or Casimir–Polder force) is a prediction of quantum electrodynamics. Classically, two uncharged parallel plates placed in a vacuum would source no electromagnetic fields and, therefore, would feel no force; quantum mechanically, they interact with virtual photons of the vacuum resulting in a net force that can be interpreted as being due to the zero-point energy of the field between the plates. This force scales as $d^{-4}$ (*d* is the distance between the plates) and is hence sub-dominant to the Newtonian force except at small separations.

This intriguing force has inspired several experiments to measure it, many of which operate at sub-mm (and even sub-micron) distances (Lamoreaux and Buttler 2005; Lambrecht and Reynaud 2011). A chameleon force (per unit area) between the two plates would scale as (Mota and Shaw 2007; Brax et al. 2007b; Brax and Davis 2015)

4.1 $$\begin{aligned} \frac{F_\mathrm{cham}}{A}\propto d^{-\frac{2n}{n+2}}, \end{aligned}$$

which always scales with a power $\ge -4$ (the bound is saturated when $n=-4$). This would dominate over the Casimir force at large separations and, therefore, the absence of any deviation from the Casimir prediction can constrain chameleon models.

In practice, it is difficult to keep the plates perfectly parallel, and very smooth plates are required for high-precision results. A more convenient scenario is the case where one of the plates is replaced by a sphere whose radius is larger compared with the separation. In this case, the Casimir force scales as $d^{-3}$ and the chameleon force would scale as

4.2 $$\begin{aligned} \frac{F_\mathrm{cham}}{A}\propto d^{\frac{2-n}{n+2}}. \end{aligned}$$

Again, this power is always $\ge -3$.

The current generation of Casimir force experiments place strong constraints on $n=-4$ and $n=-6$ chameleon models when $\varLambda _c$ is fixed to the dark energy scale. The constraints on other models are not presently competitive with other experiments discussed in this review. The next generation of experiments will use larger separations where the chameleon force is more pronounced (Lambrecht et al. 2005; Lamoreaux and Buttler 2005) so more stringent constraints on a broader class of models are expected.

Interestingly, experiments such as these can be adapted to the chameleon’s unique properties because one can vary the density of the partial vacuum inside the chamber where the experiment operates. By changing the pressure of the ambient gas, one can look for a density-dependent change in the force, which would be a smoking gun of chameleon models (Brax et al. 2010b; Almasi et al. 2015).

At the present time, Casimir force experiments have not been applied to symmetron models, mainly due to the lack of any theoretical calculations of the symmetron force between objects of different geometries.

#### Levitated microspheres

A recent addition to the fifth-force hunter’s arsenal, optically-levitated microspheres are capable of probing forces  (Geraci et al. 2010). The spheres have radii of $\mathcal {O}(\upmu \mathrm {m})$ and, in the context of chameleon models, they would, therefore, be unscreened when $\varLambda _c\ge 4.6$ meV (a factor of two above the dark energy scale). The spheres are held in an upward pointing laser beam trap by virtue of radiation pressure so as to counteract the Earth’s gravity; any anomalous motion would then be due to non-gravitational interactions. In the case of chameleon models, a microsphere held in a chameleon gradient would experience a additional force given by

4.3 $$\begin{aligned} F=\lambda \left( \frac{\rho }{M_\mathrm{c}}\right) \int _\mathrm{sphere}\, \mathrm{d}^3\mathbf {x}\frac{\partial \phi }{\partial z}, \end{aligned}$$

where *z* is the vertical direction and the sphere’s density $\rho$ is assumed to be constant. The parameter $\lambda$ is the scalar charge of the sphere. When the sphere is unscreened, which is the case for  TeV, the chameleon force is unsuppressed and $\lambda =1$. When the sphere has a thin shell, one has $\lambda <1$ and the constraints are not as stringent in this regime.

An experiment measuring forces using levitated microspheres has recently been applied to chameleon models resulting in new constraints on $n=1$ models (Rider et al. 2016); other models have yet to be considered. Constraints on symmetron models are not currently competitive with other experiments (Burrage et al. 2016b).

### Precision atomic tests

Precision atomic tests search for corrections to the structure of hydrogenic atoms by looking for non-standard perturbations to the Hamiltonian. In the case of chameleons, electrons would feel a chameleon potential in addition to the Coulomb potential given by

4.4 $$\begin{aligned} \delta H=\frac{m_e}{M_\mathrm{c}}\phi _\mathrm{N}, \end{aligned}$$

where $\phi _\mathrm{N}$ is the chameleon field sourced by the nucleus. Since the vacuum chamber shields the experiment from the effects of the external field, chameleons with strong couplings to matter can be probed by looking for the shifts in the atomic energy levels due to this perturbation. In particular, this shielding implies that the nucleus is fully unscreened so that the shifts to the lowest energy levels are (Brax and Burrage 2011)

4.5 $$\begin{aligned} \varDelta E_\mathrm{1s}&=-\frac{Zm_Nm_e}{4\pi a_0M_\mathrm{c}^2} \end{aligned}$$

4.6 $$\begin{aligned} \varDelta E_\mathrm{2s}=\varDelta E_\mathrm{2p}&=-\frac{Zm_Nm_e}{16\pi a_0M_\mathrm{c}^2}, \end{aligned}$$

where *Z* is the atomic number, $m_N$ is the nucleon mass, and $a_0$ is the Bohr radius. The potential coupling of the chameleons to photons will break the degeneracy between the 2S and 2P levels.

Presently, the 1S–2S transition in atomic hydrogen is the best constrained, having a total uncertainty of $10^{-9}$ eV (at $1\sigma$) (Jaeckel and Roy 2010; Schwob et al. 1999; Simon et al. 1980). The excellent agreement with standard atomic theory constrains the chameleon coupling4.7The effects of symmetron models on atomic transitions has yet to be investigated, although the ${\mathbb {Z}}_2$ means that the effective interaction with nucleons and electrons is higher-order i.e.

4.8 $$\begin{aligned} {\mathcal {L}}\supset m_e\frac{\phi ^2}{2M_\mathrm{s}^2}\bar{e}e, \end{aligned}$$

so that one would not expect this test to be as constraining.

### Atom interferometry

Atom interferometry is a hybridization of classical interferometric experiments and quantum mechanical double slit experiments. Atoms can be put into a superposition of two states, which travel along different paths and hence act like the arms of an interferometer. The two paths can be recombined later to produce an interference pattern that can be measured.

The atoms can be moved within the interferometer by shining laser light on them. If an atom absorbs a photon, it will be excited into a higher energy state and acquire the photon’s momentum, resulting in some linear motion. In the absence of any observation, the atom is in a superposition of the ground state (where it is stationary) and an excited state (where it is in motion). The atom can be put into a superposition of states that travel along different paths by repeating this process several times.

The probability of measuring the atom in an excited state at the output of the interferometer is a function of the difference in phases accumulated by the wave functions on the two paths. If the atom is moving in an external force field that causes some constant acceleration *a* then this probability is

4.9 $$\begin{aligned} P\,=\,\propto \cos ^2\left[ \frac{a k T^2}{\hbar }\right] , \end{aligned}$$

where *k* is the photon momentum and *T* is the duration of the experiment.

A massive object placed inside the vacuum chamber will source a gravitational field that contributes to *a*. If, in addition to this, the object sources a chameleon field then this too contributes and the probability of measuring excited atoms is sensitive to it. Since atoms placed in vacuum chambers are unscreened over a large range of the parameter space, this experiment is incredibly sensitive to chameleon and symmetron forces (Burrage et al. 2015; Burrage and Copeland 2016; Elder et al. 2016). Indeed, the first generation of atom interferometry experiments designed to test screened modified gravity was able to constrain any anomalous acceleration down to levels of $10^{-6}g$ ($g\equiv GM_\oplus /R_{\oplus }$ is the gravitational acceleration at the surface of the Earth), placing new constraints on chameleons and symmetrons that vastly reduced the viable parameter space (Hamilton et al. 2015; Burrage et al. 2016b). The current generation of experiments has constrained this further to $\lesssim 10^{-8}g$, reducing the parameter space further (Jaffe et al. 2017).

### Precision neutron tests

Neutrons are perfect objects for testing short-range gravitational physics because they are electrically neutral and are, therefore, not sensitive to electromagnetic noise such as background fields and van der Walls forces.^8^ This has motivated a recent interest in using neutrons to test chameleon models, which we summarize below. At the present time, all of the constraints derived using neutron experiments fix $\varLambda _c$ to the dark-energy scale.

#### Ultra-cold neutrons

It is possible to arrange for neutrons produced in nuclear reactors to bounce above a mirror. These neutrons interact with the Newtonian potential of the Earth leading to a quantized energy spectrum. The mirror itself could source a chameleon field, which would act as a perturbation to the neutron Hamiltonian given by (Brax and Pignol 2011; Ivanov et al. 2013)

4.10 $$\begin{aligned} \varDelta H=\frac{m_N}{M_\mathrm{c}}\phi =\frac{2.2\mathrm {\ keV}^2}{M_\mathrm{c}}\left( \frac{z}{82\,\upmu \mathrm {m}}\right) , \end{aligned}$$

where *z* is the distance above the mirror. If this perturbation were large enough, new bound states would appear in the spectrum. No such states have been observed by a qBounce experiment at the Institut Laue–Langevin in Grenoble, which immediately places a new constraint (Brax and Pignol 2011)

4.11 $$\begin{aligned} M_\mathrm{c}>10^4\mathrm {\ TeV}. \end{aligned}$$

Away from this regime, the perturbation (4.10) leads to a shift in the energy levels. This can be probed using resonance spectroscopy, the most constraining transition being $|3\rangle \rightarrow |1\rangle$. The absence of any observed shift leads to the stronger constraint (Jenke et al. 2014)

4.12 $$\begin{aligned} M_\mathrm{c}>1.7\times 10^6\mathrm {\ TeV} \end{aligned}$$

for $n=1$. In this review, we use the most up to date (at the time of writing) constraints given in Cronenberg et al. (2015).^9^

Bouncing neutron techniques have not yet been applied to symmetron models. The effective interaction for these models would be

4.13 $$\begin{aligned} m_N\frac{\phi ^2}{M_\mathrm{s}^2}\bar{n}n, \end{aligned}$$

and so one may expect a similar issue to testing symmetrons using precision atomic tests i.e. the higher-order nature of the interaction means that it would be naturally suppressed, leading to weaker constraints than chameleons.

#### Neutron interferometry

In an analogous manner to optical interferometry, a coherent beam of neutrons can be split and later recombined to produce interesting interference patterns (Pokotilovski 2013; Brax et al. 2013c). A mono-silicone crystal plate can be used for this purpose.

The proposal for testing chameleons using this technique is to introduce a cell composed of two parallel plates into the path one of the beams. A chameleon profile will develop between the two plates leading to a phase shift for the neutrons given by (Brax et al. 2013c; Brax 2014)

4.14 $$\begin{aligned} \delta \varphi =\frac{m_N^2}{\hbar ^2kM_\mathrm{c}}\int _{-d}^d\phi (x)\, \mathrm{d}x, \end{aligned}$$

where *x* is the horizontal direction and the plates are located at $x\,=\,\pm \,d$. This phase shift is maximum if the plates are in vacuum (or, rather, a partial vacuum) but diminishes if one were to inject gas at a higher density due to the suppression of the chameleon field. Such an experiment has been performed by two groups (Lemmel et al. 2015; Li et al. 2016), who report consistent bounds in the range

4.15 $$\begin{aligned} M>10^7\text {--}10^8 \mathrm {\ TeV} \end{aligned}$$

for models with $1\le n\le 6$, with stronger bounds being obtained for lower *n*.

### Astrophysical tests

In this section, we describe tests of chameleon and symmetron models using astrophysical objects. In many cases, the constraints are phrased in terms of $\chi _0$ and $\beta (\phi _0)$ and so the specific model is not important. We will not include bounds from binary pulsars since they are uncompetitive and subject to astrophysical uncertainties to do with the screening level of the Milky Way (Brax et al. 2014; Zhang et al. 2017).

#### Distance indicator tests

Determining the distance to astrophysical objects is a notoriously difficult task because only the flux of emitted photons, can be measured. Since this depends on both the distance and the absolute luminosity of the source via

4.16 $$\begin{aligned} F=\frac{L}{4\pi d^2}, \end{aligned}$$

some knowledge of the luminosity *L* is needed to infer the distance. Distance indicators are objects with some intrinsic or empirical relation between their luminosity and other observable properties. One famous example are type-Ia supernovae, where the luminosity can be found by fitting their light curve, making them standard candles.

In the context of modified gravity, it is possible that the relation used to determine the luminosity is sensitive to gravitational physics. If the relation has been calculated using general relativity, or has been determined empirically using local (screened) observations, then it will give incorrect distances when applied to unscreened galaxies. In contrast, relations that are insensitive to the theory of gravity will always give the correct distance. Comparing how well different distance estimates to theoretically unscreened galaxies agree can therefore yield new constraints.

One robust distance indicator that is not sensitive to screened modified gravity is the tip of the red-giant branch (TRGB). Low-mass post-main-sequence stars () in the process of ascending the red-giant branch (RGB) consist of an isothermal helium core surrounded by a thin hydrogen-burning shell. The hydrogen in this shell is continually processed into helium that is deposited onto the core, causing its temperature to rise steadily as the RGB is ascended. When the temperature is sufficiently high, the triple-$\alpha$ process (core helium burning) can proceed efficiently, at which point the star moves to the asymptotic giant branch in a very short time-scale. This leaves a visible discontinuity in the I-band. The discontinuity occurs at fixed luminosity [$I=4.0\pm 0.1$, the error is due to a very weak metallicity dependence (Sakai 1999; Freedman and Madore 2010; Beaton et al. 2016)], making the TRGB a standard candle. Importantly, the physics of the helium flash is set by nuclear physics and is non-gravitational in origin, elucidating our earlier assertion that this distance indicator is insensitive to modified gravity.^10^

Cepheid variable stars are distance indicators that are sensitive to modified gravity. With masses between 4 and $10M_\odot$, these stars enter a phase where their structure is dominated by semi-convection—a convective process driven by inverse-gradients in the chemical composition—shortly after ascending the RGB, resulting in large temperature increases with a relatively small change in luminosity. This results in so called *blue loops* in the Hertzprung–Russell (or color-magnitude) diagram. Whilst traversing the blue loop, the star crosses the instability strip where it is unstable to pulsations due to the presence of a layer of doubly-ionized helium.^11^ Cepheids pulsate with a well-measured period-luminosity relation (PLR), where the period $\varPi \propto \sqrt{R^3/G{\mathcal {M}}}$. This relation is, therefore, different in unscreened galaxies and, in particular, if one applies the locally measured formula to an unscreened galaxy one under-estimates the distance by a factor

4.17 $$\begin{aligned} \frac{\varDelta d}{d}\approx -0.3\frac{\varDelta G}{G}. \end{aligned}$$

The screening mechanisms above can therefore be tested by comparing TRGB and Cepheid distances to unscreened galaxies. Jain et al. (2013a) have done precisely this for a sample of 25 galaxies taken from the screening map (Cabre et al. 2012). They also compared distances to a similar sample of screened galaxies as a control set. They found a similar agreement and scatter in both cases, and a $\chi ^2$-fit to both GR and modified gravity models yielded constraints^12^ in the $\chi _0$–$\beta (\phi _0)$ plane that we translate into chameleon, symmetron, and *f*(*R*) parameters in Sect. 5.

#### Rotation-curve tests

The circular velocity of objects orbiting the center of galaxies is given by

4.18 $$\begin{aligned} v_c^2=\frac{G{\mathcal {M}}_\mathrm{gal}(r)}{r^2}\left( 1+2\beta (\phi _0)\frac{Q}{{\mathcal {M}}}\right) , \end{aligned}$$

where the scalar charge *Q* is defined in Eq. (3.34) and ${\mathcal {M}}_\mathrm{gal}(r)$ is the galactic mass enclosed by *r*. If , then dwarf galaxies are unscreened but their constituent stars are not because their Newtonian potential allows them to self screen (see Table 1). Stars in unscreened dwarf galaxies therefore have $Q/{\mathcal {M}}=0$. In contrast, diffuse hydrogen gas with $\varPhi _\mathrm{N}\sim 10^{-11}$–$10^{-12}$ cannot self-screen and has $Q/{\mathcal {M}}=\beta (\phi _0)$. Assuming that the galaxy is completely unscreened, the ratio of the circular velocity of stars and gas is then

4.19 $$\begin{aligned} \frac{v_{c,\,\mathrm{gas}}}{v_{c,\,\star }}=\sqrt{1+2\beta ^2(\phi _0)}, \end{aligned}$$

implying that the galactic rotation curve measured using stellar observations will disagree with the rotation curve measured using observations of the interstellar gas. This is a direct consequence of the equivalence principle violation (i.e. $Q\ne {\mathcal {M}}$).

Measurements of the galactic rotation curves typically use either H$\alpha$ emission or the 21-cm line, both of which probe the gaseous component. An alternate but less prevalent method involves measuring the Mgb triplet lines, which are due to absorption in the atmosphere of K- and G-stars (). At present, the screening map contains six unscreened dwarf galaxies, for which both Mgb and either H$\alpha$ or 21-cm data (or both) are available. Using this, Vikram et al. (2014) have reconstructed both the gaseous and stellar rotation curves, and have used them to test the prediction (4.19) using a separate $\chi ^2$ fit for each galaxy. This has placed new constraints in the $\chi _0$–$\beta (\phi _0)$ plane, which are comparable with the Cepheid bounds.

#### Galaxy clusters

The predicted difference between the dynamical and lensing masses discussed in Sect. 3.1.2 can be tested using observations of galaxy clusters, for which there is a wealth of X-ray and weak-lensing data available. The X-ray brightness temperature is a measure of the mass of the hot gas in the intra-cluster medium, which is in hydrostatic equilibrium and hence satisfies^13^

4.20 $$\begin{aligned} \frac{\, \mathrm{d}P}{\, \mathrm{d}r} = -\frac{G{\mathcal {M}}_\mathrm{dyn}\rho }{r^2}. \end{aligned}$$

X-ray observations, therefore, probe the dynamical mass, whereas weak lensing probes the lensing mass, so comparing the two places new constraints on screening. This was first done by Terukina et al. (2014) using observations of the Coma cluster to find the new constraint $f_{R0}<6\times 10^{-5}$. Wilcox et al. (2016) subsequently applied the same methodology to a sample of 58 clusters using X-ray data from the XMM Cluster Survey and weak-lensing data from CFHTLenS to obtain further constraints on more general chameleon models.

### *f*(*R*) specific tests

In this section, we will briefly summarize tests that have been specifically designed to test the Hu and Sawicki (2007) *f*(*R*) theories discussed in Sect. 2.1.1. Note that, since these theories correspond to chameleons with $-1<n<-1/2$, many of these tests are unconstraining for more general chameleon models. Similarly, specific tests are needed to target this parameter range. Note also, that *f*(*R*) models are designed to be cosmologically relevant, and so the majority of the tests discussed here are astrophysical in nature. In what follows, we will only focus on $b=1$ ($n=-1/2$) models because the majority of tests have reported constraints for this model only. Larger values of *b* are more readily screened and so one would expect the constraints to be weaker. Note that some tests mentioned above report bounds on $f_{R0}$. We will not repeat that discussion here. A full list of constraints on $f_{R0}$ can be found in Table 1 of Lombriser (2014).

#### Solar-system bounds

One can solve the field equations sourced by the Sun to find a bound on the the value of $f_R^\mathrm{gal}=\, \mathrm{d}f(R)/\, \mathrm{d}R(\rho ^\mathrm{gal})$ (defined as $\, \mathrm{d}f(R)/\, \mathrm{d}R$ at the Milky Way density) (Hu and Sawicki 2007)4.21where $\gamma$ is the Eddington light-bending parameter in the PPN formalism. Relating the galactic density to the cosmological density ($\rho ^\mathrm{gal}=10^{-24}$ g cm$^{-3}$) one finds

4.22 $$\begin{aligned} f_{R0}<74(1.23\times 10^6)^{b-1}\left[ \frac{R_0}{\mu ^2}\frac{\varOmega _mh^2}{0.13}\right] ^{-(b+1)}, \end{aligned}$$

which gives  for $b=1$.

#### Strong gravitational lensing

Another method to probe the predicted discrepancy between the dynamical and lensing mass of an object is to use strong lensing by individual galaxies. In this case, one can use the stellar dispersion relation to calculate the dynamical mass. Smith (2009) has performed such a test for a sample of galaxies from the Sloan Lens ACS (SLACS) survey and find a constraint $f_{R0}<2.5\times 10^{-6}$.

#### Cluster density profiles

N-body simulations of *f*(*R*) gravity have repeatedly predicted an enhancement in the dark matter halo density profiles around the virial radius compared with GR (Schmidt et al. 2009a; Schmidt 2009). This is an artefact of the late-time unscreening in *f*(*R*) models. The center of the galaxy is largely unaffected because it is both screened and formed earlier when the screening was more efficient. In contrast, there is a pile-up of mass in the outer regions, which form at later times, due to the weaker screening. Lombriser et al. (2012b) has used weak lensing data for the Max-BCG galaxy cluster sample from the SDSS to probe this potential novel feature, finding a constraint $f_{R0}<3.5\times 10^{-3}$.

#### Cluster abundances

The statistics of galaxy clusters is very sensitive to the theory of gravity. For *f*(*R*) theories, the enhanced gravitational force results in a higher abundance of rare massive clusters compared with GR (Schmidt et al. 2009a) meaning the halo mass function is modified. Making quantitative theoretical predictions for this requires knowledge of physics deep within the non-linear cosmological regime and so N-body simulations and spherical collapse halo models calibrated on them are required in order to make quantitative predictions.

The first bound obtained by looking at cluster abundances yielded $f_{R0}<1.2\times 10^{-4}$ (Schmidt et al. 2009b). This was obtained by using X-ray inferred clusters in combination with a variety of different cosmological datasets available at the time. A stronger bound $f_{R0}<1.6\times 10^{-5}$ has subsequently been obtained by Cataneo et al. (2015) using a full MCMC analysis of the cluster likelihood function for updated datasets from more recent cosmological surveys.

#### Cosmic microwave background

Modifications of GR change the structure of the equations describing linear cosmological perturbations, and can hence effect the cosmic microwave background (CMB) (Zhang 2006; Song et al. 2007; Dossett et al. 2014). Updating various CMB codes to include the effects of *f*(*R*) gravity, several groups have all obtained a similar bound $f_{R0}<10^{-3}$ (Song et al. 2007; Dossett et al. 2014; Raveri et al. 2014; Cataneo et al. 2015).

#### Scalar radiation

As was first pointed out by Silvestri (2011), pulsating stars should source scalar radiation and hence lose energy over time. If too much scalar monopole radiation (which is absent in GR) is emitted, then the pulsations may quench. This was investigated by Upadhye and Steffen (2013), who found that the energy loss to monopole radiation is too weak to place any meaningful bounds. They identified another scenario whereby the scalar radiation sourced by an expanding type II supernovae could drain the kinetic energy of the expanding matter and significantly impede the expansion. This places the weak constraint $f_{R0}<10^{-2}$.

#### Redshift-space distortions

The clustering of matter can be greatly modified in *f*(*R*) cosmologies compared with GR, and this can be particularly pronounced in redshift space (Jennings et al. 2012; Bose and Koyama 2016, 2017). The possibility of testing this was first investigated by Yamamoto et al. (2010), who examined a sample of luminous red galaxies (LRGs) from the SDSS to find a bound $f_{R0}<10^{-4}$. A more recent study, combining redshift-space distortion observations with other cosmological datasets, found the stronger bound $f_{R0}<2.6\times 10^{-6}$ (Xu 2015).

### Tests of the coupling to photons

In this section, we summarize experimental tests of the coupling to photons discussed in Sect. 2.3. We will restrict our attention to chameleon models, for which the coupling to photons has been widely studied. Extending these constraints to other models with screening remains a topic for future work.

#### PVLAS

The PVLAS experiment (Zavattini et al 2006) studied the polarisation of light propagating through a magnetic field. The presence of an axion, or axion-like particle coupled as in Eq. (2.35) would mean that, in the presence of a magnetic field, one polarisation of the propagating photon can convert into the scalar particle and vice versa. The second polarisation will propagate through unimpeded (Raffelt and Stodolsky 1988). This induces rotation and ellipticity into the polarisation of the incoming laser beam. The PVLAS experiment bounded the induced rotation to be less than $1.2\times 10^{8}$ rad at 5 T and $1.0\times 10^{8}$ rad at 2.3 T, and the induced ellipticity to be less than $1.4 \times 10^{8}$ at 2.3 T. This constraints the coupling strength $M_{\gamma }$ of a light axion-like particle.

In such experiments chameleon particles behave very differently to standard axion-like particles, precisely because of their density dependent mass. If standard axion-like particles were produced in PVLAS, they would pass through the walls at the end of the vacuum chamber without interacting and so leave the experiment. For a chameleon to pass through the wall, the chameleon particle must have enough energy that it can adjust its mass to the higher value needed for it to exist inside the wall. If it does not have this energy, it is instead reflected from the wall and back into the vacuum chamber (Brax et al. 2007a, c). This leads to a large ratio of the rotation to the ellipticity of the polarisation which is a unique signal of chameleon models. For a chameleon with a potential $V(\phi )=(2.3 \times 10^{-3} \mathrm {\ eV})^5/\phi$, and assuming the coupling to photons is the same as the coupling to other matter fields, the results of the PVLAS experiment constrain $M_\mathrm{c}=M_{\gamma }> 2\times 10^6 \mathrm {\ GeV}$.

#### GammeV-CHASE

A second commonly used experimental design to look for axion-like particles, light-shining-through-walls, also needs to be modified in order to search for chameleon particles. Experiments searching for standard ALPs rely on the ability of ALPs to pass through walls which are impermeable to photons. Light is shone into a cavity across which a magnetic field is applied. A wall is then placed in this cavity; in the absence of ALPs, no light would be seen on the far side of the wall. But if a photon converts into an ALP before hitting the wall this ALP can pass through and then may reconvert into a photon on the far side of the wall.

As discussed in the previous subsection, chameleon ALPs cannot pass through walls in the way that standard ALPs do, and so light-shining through walls experiments cannot constrain chameleons. However, this inability to pass through walls can be developed into a new type of experiment specifically designed to look for chameleons; these are known as after-glow experiments (Gies et al. 2008; Ahlers et al. 2008). The basic design of the experiment is to shine a laser beam into a vacuum chamber across which a magnetic field is applied. If there is a non-zero probability of the photons converting into chameleons, then the number of chameleons trapped inside the chamber (because they cannot pass through the walls) will increase the longer the laser beam is on. The laser is then turned off, but the magnetic field is left on. Then the chameleons can reconvert into photons, leading to a detection of light, after the laser has been turned off.

This experiment was successfully performed by the GammeV collaboration, and was known as GammeV-CHASE (GammeV CHameleon Afterglow SEarch) (Upadhye et al. 2010). Constraints were placed on values of the chameleon coupling to photons, as a function of the effective chameleon mass in the chamber (Chou et al. 2009). This mass depends on the choice of the chameleon potential and the strength of the coupling to other matter fields. For the lightest chameleons inside the vacuum chamber, GammeV-CHASE constrains the coupling to photons to be $M_{\gamma }>3 \times 10^7 \mathrm {\ GeV}$ (Steffen et al. 2010; Upadhye et al. 2012b). The constraints weaken if the effective mass of the chameleon is above $10^{-3} \mathrm {\ eV}$.

The modelling of how the chameleon behaves inside the experiment requires care. Whilst a semi-classical approximation would predict that the chameleon bounces off the walls of the vacuum chamber unchanged, considering the chameleons as fluctuations in a quantum field opens up the possibility that the non-trivial self interactions of the chameleon field could allow a chameleon particle to fragment into a number of lower energy chameleons as it hits the wall. This was shown not to be a significant effect in the GammeV-CHASE experiment for the benchmark potentials $V(\phi )= \lambda \phi ^4$ and $V(\phi )=\varLambda ^5/\phi$ (Brax and Upadhye 2014). However, for steeper potentials this effect will start to become relevant.

#### ADMX

Axion Dark Matter eXperiment (ADMX), is another experiment aiming to detect axions and axion-like particles through the Primakov effect (Asztalos 2010, 2004). However, in this case, the axions come from outside the experiment, and are hypothesised to be responsible for the dark matter in our galaxy (Sikivie 1983). This set up has been used to constrain chameleon theories using the same afterglow effect discussed above (Rybka et al. 2010), but using microwave photons trapped in a cavity instead of laser light. The experiment excluded couplings $5 \times 10^3 \mathrm {\ GeV}< M_{\gamma }< 1 \times 10^9 \mathrm {\ GeV}$ for effective chameleon masses in the cavity $\sim 1.95\, \upmu \mathrm{eV}$.

#### CAST

The CERN Axion Solar Telescope (CAST) experiment searches for axions produced in the Sun, by looking for their reconversion into photons in the bore of a decommissioned LHC magnet (Zioutas et al. 2005). Results from this search can be applied to chameleons, if they are also produced in the Sun. At the particle level, the processes that produce chameleons are the same as those that produce scalar axion-like particles, but determining the total flux of chameleons from the Sun requires taking into account the added complication that the mass of the chameleon field varies with the density of the solar medium (Brax and Zioutas 2010).

CAST has not yet detected a signal from the Sun, and so bounds can be placed on the chameleon couplings. They exclude photon couplings $M_{\gamma } \ge 2.6 \times 10^7 \text{ GeV }$, for a range of couplings to matter $10^{12} \mathrm {\ GeV} \le M_\mathrm{c}\le 10^{18} \mathrm {\ GeV}$, assuming that the bare chameleon potential is $V(\phi ) =(10^{-3}\mathrm {\ eV})^5/ \phi$ (Anastassopoulos et al. 2015).

There are also proposals by the CAST collaboration to detect solar chameleons using a novel force sensor (Baum et al. 2014). While chameleons may be produced in the Sun due to the coupling to photons, the detection mechanism itself does not rely on the coupling in Eq. (2.35). The detection relies on having a force sensor sufficiently sensitive that it can measure the chameleon radiation pressure (Karuza et al. 2016), which comes about as the chameleons emitted from the Sun bounce off the sensor, for the same reason that chameleons are reflected from the walls of vacuum chambers, the chameleon particle does not have enough energy to adjust its mass sufficiently to pass through the membrane of the sensor.

#### Collider constraints

The collider constraints on chameleon models can also be extended to include the coupling to photons in Eq. (2.35). This leads to additional loops, which should be inserted into the diagrams, and allows for additional production and decay processes which should be included. Analysis of precision electro-weak data from LEP constrains $M_{\gamma }\gtrsim 10^3\mathrm {\ GeV}$ (Brax et al. 2009).

#### Galactic and extra-galactic constraints

The effects of the chameleon on light propagating through magnetic fields, originating in the interaction of Eq. (2.35), can also be relevant to astrophysical observations. For many observations, light from distant sources has to propagate through galactic, intra-cluster, or extra-galactic magnetic fields in order to reach us. Whilst the magnetic fields strengths are much lower than those achievable in the laboratory, they extend over much larger distances, meaning that the astrophysical constraints can in principle be more stringent that those achieved in the laboratory. They do, however, come with much larger uncertainties around the initial luminosity of the source, the polarisation of the light it emits, and over the structure of the magnetic fields. Astrophysical magnetic fields also display much more structure than the coherent magnetic fields used in laboratory, which adds to the complexity of the calculations.

In Burrage et al. (2009b), it was shown that chameleons coupled to photons can induce both linear and circular polarisation into light from stars. As long as the chameleon mass is smaller than the local plasma density, then it can be neglected in these calculations, meaning that the constraints are largely model independent as long as the chameleon is light on astrophysical scales. Within the galaxy this requires $m_{\phi } <1.3 \times 10^{-11} \mathrm {\ eV}$. From measurements of the polarisation of galactic stars, expected to be largely unpolarized initially, the bound $M_\gamma > 1.1 \times 10^9 \mathrm {\ GeV}$ was derived. Assuming the magnetic field strength of the intergalactic medium is $B\approx 3\, \upmu \mathrm {G}$ and the coherence length is $20 \mathrm {\ pc}$. The polarisation of light from the Crab nebula, type Ia supernova, high-redshift quasars, gamma-ray bursts, and the CMB was also analysed but the bounds were weaker than those from observations of stars.

Looking for the depletion in luminosity of astrophysical sources from photons converting into chameleons is difficult because there is, generally, no way of determining the intrinsic luminosity of the source. However, for some astrophysical objects, correlations have been observed between the luminosity of the source and a second observable that should not be affected by the coupling to chameleons. The best constraints of this form on chameleons come from looking at Active Galactic Nuclei (AGN), where the X-ray luminosity at $2 \mathrm {\ keV}$ is observed to be tightly correlated with the optical luminosity at $5 \mathrm {\ eV}$ (Steffen et al. 2006; Young et al. 2009). Similar luminosity relations exist for blazars and gamma ray bursts, but these give rise to weaker constraints. As the probability of a photon converting into a chameleon increases with the frequency of the photon, the effects of the chameleon on the X-ray luminosity of the AGN can be significant, whilst the effects on the optical luminosity remain small. Therefore, the luminosity relation can be used to constrain the chameleon (Burrage et al. 2009a), with the current best constraint $M_{\gamma }\gtrsim 10^{11}\,\mathrm {\ GeV}$ assuming, again, that the chameleons are sufficiently light, $m_{\phi }<10^{-12}\,\mathrm {\ eV}$, on astrophysical distance scales that the effects of their mass are negligible (Burrage et al. 2009a; Pettinari and Crittenden 2010).

The conversion of photons into chameleons also will increase the opacity of the universe at high frequencies. In Avgoustidis et al. (2010), tests of the distance duality relation, which relates luminosity distance and angular diameter distance to sources, were used to derive constraints on cosmic opacity. This can be viewed as a test of chameleons because depletion of photons from the source will change the luminosity distance, whilst leaving the angular diameter distance unaffected. Constraints are currently not competitive with those from starlight polarisations, but should be expected to improve significantly with new data from upcoming cosmological surveys.

Light from the cosmic microwave background also passes through magnetic fields on its way to us, although constraints from CMB intensity and polarisation data are difficult to apply because of our lack of knowledge about primordial magnetic fields (Schelpe 2010). Knowledge of the magnetic fields of localised objects, such as the Coma cluster, mean that constraints can be obtained from measurements of the Sunyaev–Zel’dovich (SZ) effect. The SZ effect is the distortion of the CMB spectrum by inverse Compton scattering of high-energy electrons. The effect of converting photons into chameleons in the cluster’s magnetic field, also depletes the expected photon number, but with a very different frequency dependence. Knowledge of the Coma cluster’s magnetic fields leads to the constraint $1.1 \times 10^9 \mathrm {\ GeV} \lesssim M_{\gamma }$ (Davis et al. 2011).

**Table 2:** Summary of present tests of chameleon and symmetron theories

Test	Chameleons	Symmetrons
Eöt-Wash	✓	✓
Casimir force	✓	✗
Microspheres	✓	✗
Precision atomic tests	✓	✗
Atom interferometry	✓	✓
Cold neutrons	✓	✗
Neutron interferometry	✓	✗
Distance indicators	✓	✓
Rotation curves	✓	✓
Cluster lensing	✓	✗

**Fig. 4:**
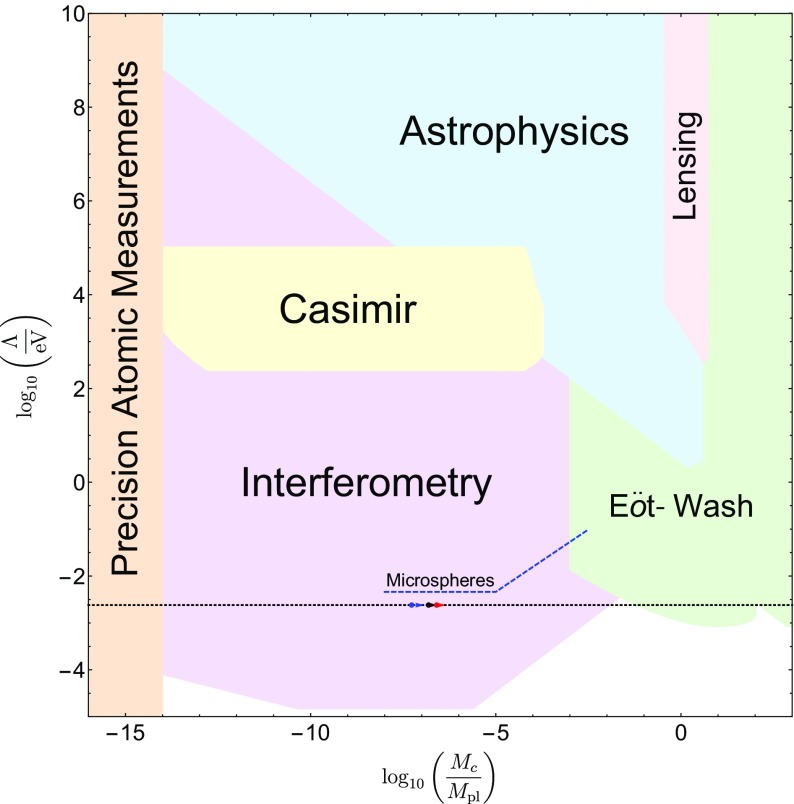
Current bounds on the parameters $M_\mathrm{c}$ and $\varLambda$ for $n=1$ chameleon models. The regions excluded by each specific test are indicated in the figure; the region labelled astrophysics contains the bounds from both Cepheid and rotation curve tests. The dashed line indicates the dark energy scale $\varLambda =2.4$ meV. The black, red, and blue arrows show the lower bound on $M_\mathrm{c}$ coming from neutron bouncing and interferometry. The blue corresponds to the bounds of Lemmel et al. (2015) and the red to the bounds of Li et al. (2016)

**Fig. 5:**
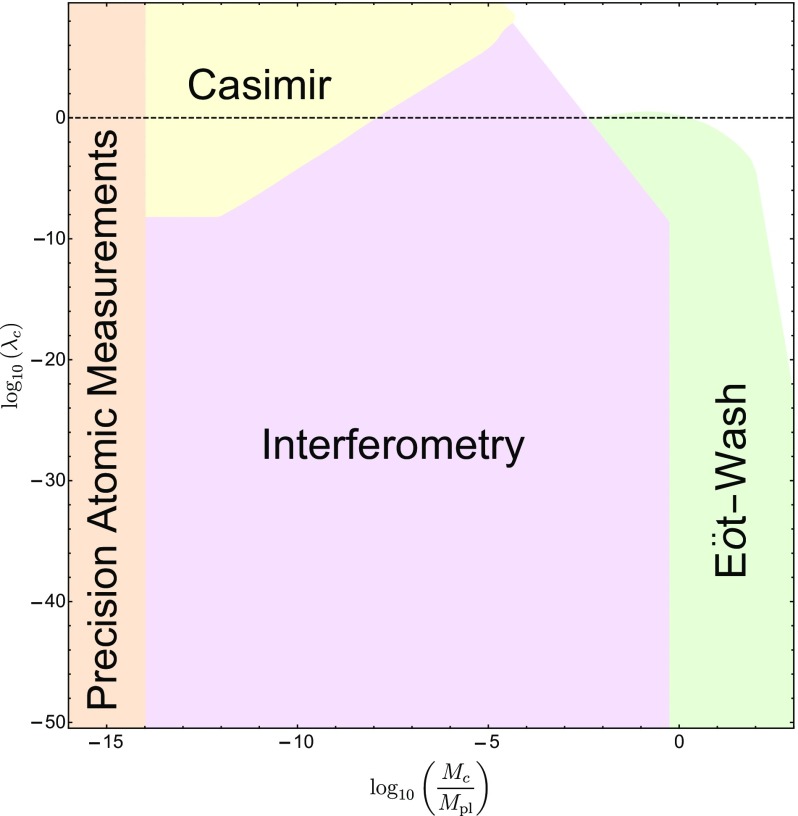
Current bounds on the parameters $M_\mathrm{c}$ and $\varLambda$ for $n=-4$ chameleon models. The regions excluded by each specific test are indicated in the figure. Comparing Eqs 2.16 with 2.17 reveals that $\lambda _\mathrm{c} = (\varLambda /\varLambda _\mathrm{DE})^4$ and so the values of $\lambda _\mathrm{c}$ plotted here cover the same range of $\varLambda$ as Fig. 4. The black dashed line at $\lambda _\mathrm{c}=1$ therefore corresponds to the dark energy scale $\varLambda =\varLambda _\mathrm{DE}$

**Fig. 6:**
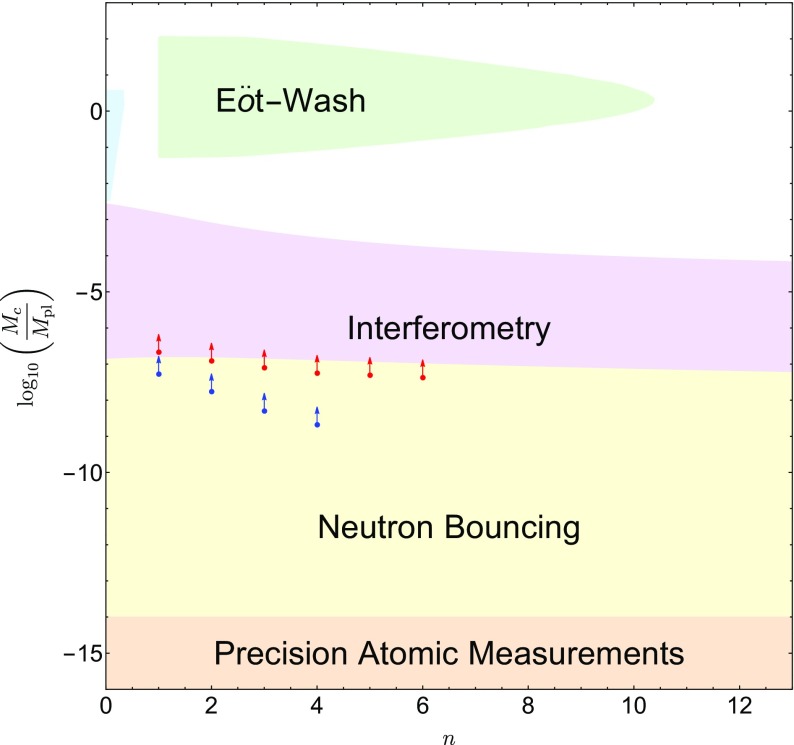
Current bounds on the parameters *n* and $M_\mathrm{c}$ when $\varLambda$ is fixed to the dark energy scale $\varLambda _\mathrm{DE}$ and $n>0$. The regions excluded by each specific test are indicated in the figure. The blue region corresponds to astrophysical tests, which includes both Cepheid and rotation curve tests. The blue and red arrows indicate the lower bounds coming from the neutron interferometry experiments of Lemmel et al. (2015) and Li et al. (2016) respectively

**Fig. 7:**
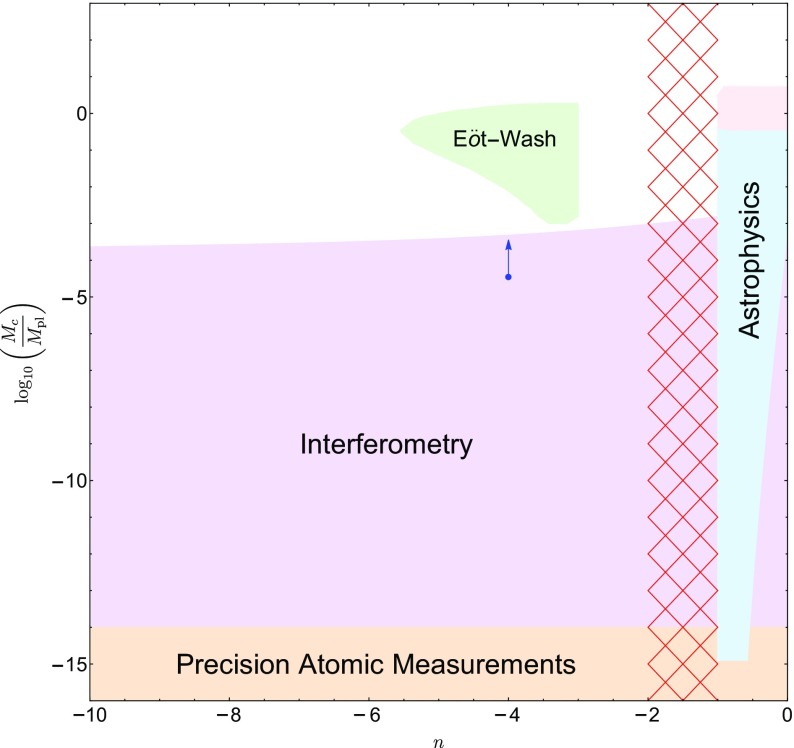
Current bounds on the parameters *n* and $M_\mathrm{c}$ when $\varLambda$ is fixed to the dark energy scale $\varLambda _\mathrm{DE}$ and $n<0$. The red hashed region indicates values of *n* where the model is not a chameleon, and the reader is reminded that only negative even integers are chameleons. The regions excluded by each specific test are indicated in the figure; the region labelled astrophysics contains the bounds from both Cepheid and rotation curve tests. The blue arrow indicates the lower bound coming from the neutron interferometry experiment of Lemmel et al. (2015)

### Summary of tests

Here, we briefly summarize the tests that have been used to probe screened modified gravity to date. The summary is given in Table 2; we do not include *f*(*R*)-specific tests, because they do not carry over to more general models.

## Constraints

In this section, we convert the constraints discussed in the previous section into a single and familiar parametrization and combine them to show the presently allowed parameter ranges.

### Chameleon constraints

The current bounds on chameleon models are shown below. We cover the two most commonly studied models $n=1$ (Fig. 4) and $n=-4$ (Fig. 5). In these cases, we plot $\varLambda$ versus $M_\mathrm{c}$. Furthermore, many experiments focus on the case $\varLambda =\varLambda _\mathrm{DE}=2.4$ meV (the dark energy scale) and so for this choice we plot $M_\mathrm{c}$ versus *n* for both positive (Fig. 6) and negative *n* (Fig. 7).

#### *f*(*R*) constraints

**Fig. 8:**
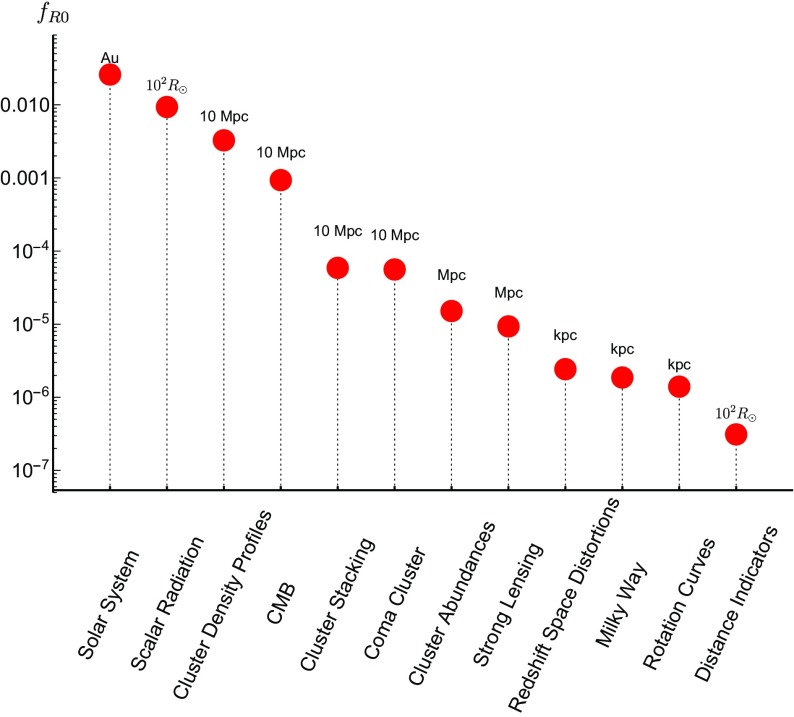
Constraints on $f_{R0}$ for $b=1$ Hu and Sawicki *f*(*R*) models (see Eq. (2.19)). The red dots indicate the upper limit for the specific test given on the *x*-axis and the points are labelled by the typical distance scale associated with the relevant test

We show the current constraints on the Hu and Sawicki $b=1$ *f*(*R*) model (2.19) in Fig. 8. The *x*-axis labels each specific test and the *y*-axis shows the resultant upper limit on $f_{R0}$. It is common to express constraints on $f_{R0}$ showing the length scale on which they were obtained (e.g., Lombriser 2014). Whilst complementary tests on all scales are crucial consistency checks of the theory, it is important to note that this length is not a new parameter appearing in the theory, and that it is the same parameter $f_{R0}$ being constrained no matter the test or the length scale that it probes. For this reason, we have included the typical length scale for each test in the figure.

The point labelled “Milky Way” is not derived from any specific test and is simply the statement that the $f_{R0}$ should be smaller than the Newtonian potential of the Milky Way. One does not need to impose this *a priori* because it is not clear whether or not the Milky Way is screened by the local group; we include it here for completeness, and to make contact with those parts of the literature that take this constraint as given.

#### Constraints on the coupling to photons

**Fig. 9:**
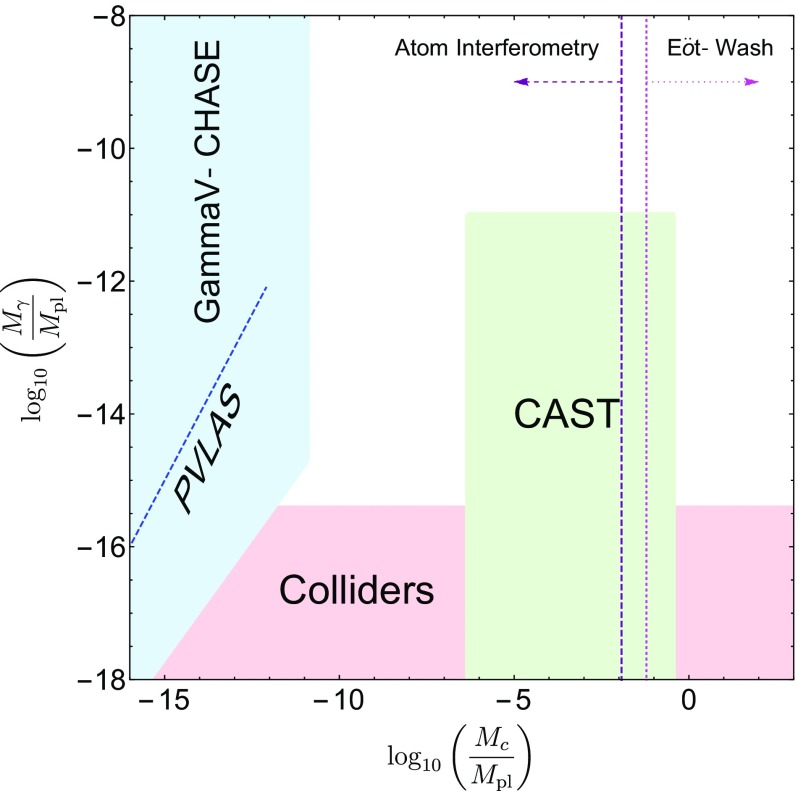
Current constraints on the chameleon coupling to photons, $M_\gamma$, for $n=1$ models with $\varLambda$ set to the dark energy scale. The bounds coming from each specific test are indicated in the figure

The constraints on the coupling to photons are shown in Fig. 9. We only show constraints for $n=1$ models since many experiments only report bounds for these models at the present time. Furthermore, many of the experiments restrict to the case $\varLambda =\varLambda _\mathrm{DE}=2.4$ meV and so we do the same here. The results from ADMX are not included since they are presented in terms of $m_\mathrm{eff}$ rather than the fundamental parameters. One could convert the constraints into the $M_\mathrm{c}$–$M_\gamma$ plane, but this depends on the geometry and densities of the experimental apparatus, which are not sufficiently well known. Similarly, we do not include astrophysical bounds due to the need to make assumptions about the strength of magnetic fields and the value of the ambient density.

### Symmetron constraints

**Fig. 10:**
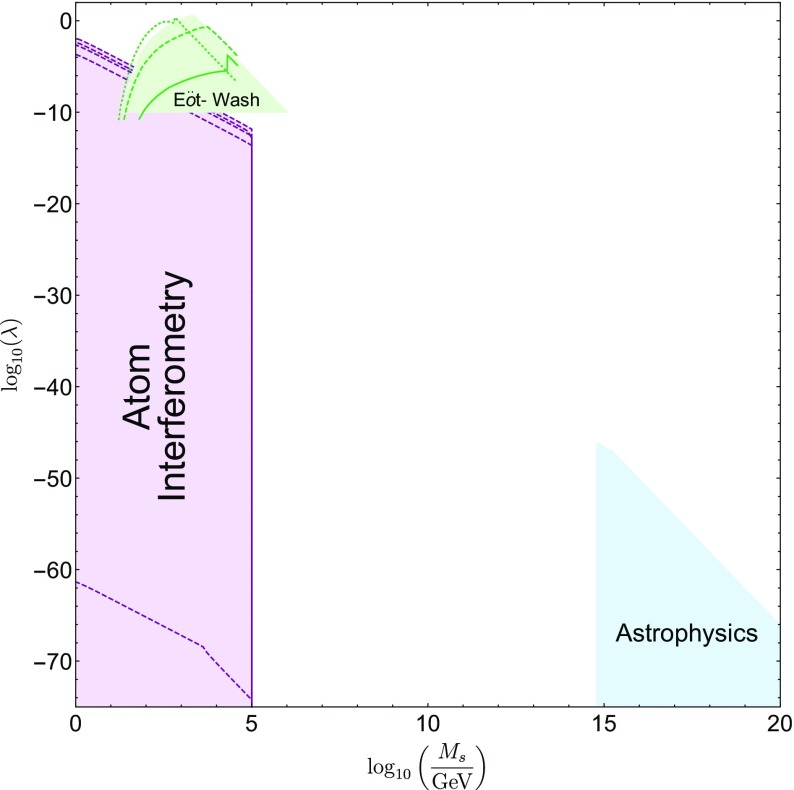
The current bounds on the symmetron parameters $M_\mathrm{s}$ and $\lambda$. The region of parameter space excluded by each specific test is indicated in the figure. The Eöt-Wash region corresponds to $\mu =2.4$ meV; the outlines for values $\mu =\{10^{-4},\,10^{-3},\,10^{-2}\}$ eV are shown by the solid, dashed, and dotted green lines respectively. The atom interferometry lines correspond to the regions excluded for $\mu =\{10^{-4},\,10^{-4.5},\,10^{-5},\,10^{-5},\, 2.4\times 10^{-3}\}$ eV from top to bottom respectively, the latter value corresponding to the dark energy scale. The astrophysical bounds are insensitive to the value of $\mu$ for the values considered here

The current bounds on the symmetron parameters $M_\mathrm{s}$ and $\lambda$ are shown in Fig. 10 for some commonly studied values of $\upmu$ indicated in the caption.

## Conclusions and outlook

Chameleon and symmetron models have been a paragon for viable, interesting, and relevant infrared modifications of general relativity for over a decade. The screening mechanism has resulted in theories of gravity that are perfectly consistent with general relativity’s predictions in the solar system, but are yet falsifiable using novel approaches such as astrophysical phenomena in distant galaxies, as well as specifically targeted laboratory searches. In many cases, these models may be relevant on linear (and non-linear) cosmological scales.

In this review, we have surveyed the omnibus of literature providing constraints and have translated them into a single parametrization in order to assess the current viability of the models. The main results are presented in Figs. 4, 5, 6, 7, 8, 9 and 10, which can be summarized as follows:

- $n=1$ and $n=-4$ chameleon models (two of the most commonly studied) are tightly constrained but there is a large parameter space remaining for $n>1$ and $n<-4$ when $\varLambda$ is fixed to the dark energy scale. Away from this, the constraints are not as strong. In many cases, this is because bounds on other models are not reported.
- Symmetron models are well-constrained by astrophysical probes and atom interferometry but there is a lack of theoretical work translating the bounds from existing experimental results into symmetron constraints. This has resulted in a desert separating astrophysical and laboratory tests [this could be filled in partially by constraints from future space-based tests of relativistic gravitation (Sakstein 2017)].
- The coupling of chameleons to photons for $n=1$ models is tightly constrained and there is only a narrow window remaining. The coupling of symmetrons to photons and chameleon models with $n\ne 1$ has yet to be explored.
- Hu and Sawicki *f*(*R*) models (Hu and Sawicki 2007) are well-constrained for $b=1$ but, presently, there are not enough reported bounds on larger values to make a meaningful comparison. For $b=1$ the bounds on $f_{R0}$ are at the $10^{-7}$ level. In theory, $10^{-8}$ would be achievable with better statistics; below this, dwarf galaxies begin to become screened and higher-precision tests are necessary.
- At the present time, the environment-dependent dilaton, which screens in a distinct manner from chameleon and symmetron models, has not been studied sufficiently in the context of laboratory and astrophysical tests to produce any meaningful constraints.

### Prospects for future bounds

We end by discussing the prospects for future tests of screened modified gravity.

#### Laboratory tests

As new experimental techniques are been developed, and existing ones are improved we can expect bounds on chameleon and symmetron models of screening to continue to improve. It is to be expected that this will be a combination of the reinterpretation of experimental results obtained when searching for other types of new physics, and a smaller number of experiments dedicated to directly searching for screening.

It is difficult to imagine that a single experiment could cover all of the remaining chameleon and symmetron parameter space, and so ideally a combination of techniques and searches are needed in order to fully rule out the possibility that screened scalars exist in our Universe.

#### Astrophysical tests

Astrophysical objects show strong deviations from GR when the Newtonian potential $\varPhi _\mathrm{N}<\chi _0$ ($\sim f_{R0}$ for *f*(*R*) theories). Given that current bounds place , the only objects in the Universe with a low enough Newtonian potential to exhibit novel effects are dwarf galaxies located in voids, and several tests using such galaxies have been proposed.

The rotation-curve test described in Sect. 4.5.2 suffers from a lack of unscreened galaxies, and a larger sample would improve the constraints. Future and upcoming data releases, in particular SDSS-MaNGA, can provide a larger sample size that would significantly improve the bounds. Additional tests, such as the warping of galactic disks due to equivalence principle violations have been proposed (Jain and VanderPlas 2011), although a test using SDSS optical and ALFALFA radio observations did not yield any bounds on the model parameters (Vikram et al. 2013). Future radio surveys such as VLT may be more fruitful.

Finally, N-body simulations are uncovering a variety of novel phenomena exhibited by chameleons on non-linear cosmological scales (Jain et al. 2013b). Many of these are clear smoking-gun signals that could be measured with upcoming peculiar velocity and galaxy redshift surveys (Hellwing et al. 2014).

#### Tests of the coupling to photons

The increase in interest in axions and axion-like particles as dark matter candidates has lead to a series of proposals and experiments aimed at further constraining these particles which, in many cases, focus on their interactions with photons. These experiments present an exciting opportunity for new constraints on theories with screening, but the details of how powerful these constraints can be remain to be worked out.

## Footnotes

1 Note that one could consider a more general theory where each particle species *i* is conformally coupled to a different metric $\tilde{g}^{(i)}_{\mu \nu }=A_i^2(\phi )g_{\mu \nu }$ although we will not consider such theories here since they are not well-studied in the context of screened modified gravity. An even more general metric includes disformal terms $\tilde{g}_{\mu \nu }=A(\phi )g_{\mu \nu } +B(\phi )\partial _{\mu }\phi \partial _{\nu }\phi$ (Bekenstein 1993). Constraints on disformal couplings to matter can be found in Brax and Burrage (2014), Sakstein (2014b), Sakstein (2015), Ip et al. (2015) and Sakstein and Verner (2015).

2 There are three commonly used densities in the literature: the Jordan frame density $\tilde{\rho }=-\tilde{T^{0}_0}$, the Einstein frame density $\rho =-T^0_0=A^6(\phi )\tilde{\rho }$, and the ‘conserved Einstein frame density’ $\rho _\mathrm{conserved}=A(\phi )\rho$. The Jordan frame density is the result of statistical physics calculations and it is in this frame that one may specify an equation of state. The Einstein frame density is what arises naturally in Eq. (2.6) as a result of varying the action (2.1) and the conserved density is a quantity that is useful in cosmological contexts (Khoury and Weltman 2004a; Hui et al. 2009; Brax et al. 2012a; Brax and Davis 2012; Brax et al. 2012b; Sakstein 2014a). In particular, the conserved density satisfies a conservation equation that makes the cosmological equations look similar to those of GR. Since this review is concerned with experimental tests of chameleon theories, we have opted to work with the Einstein frame density. At the Newtonian level (weak-field limit), these densities are equivalent (Hui et al. 2009; Sakstein 2014a) and so the choice is somewhat arbitrary, but we note that one must work with the Jordan frame pressure and density if one is interested in compact objects such as neutron stars (Babichev and Langlois 2010; Minamitsuji and Silva 2016; Babichev et al. 2016; Sakstein et al. 2017; Brax et al. 2017). We will not consider such objects here.

3 Several definitions of the effective potential exist in the literature. If one uses the conserved Einstein frame density then one has $V_\mathrm{eff}(\phi )=V'(\phi )+\rho A(\phi )$ (Khoury and Weltman 2004a; Sakstein 2014a). Furthermore, one often sees the effective potential written as $V_\mathrm{eff}(\phi )=V'(\phi )+(A(\phi )-1)\rho$ (using the conserved Einstein frame density). This is motivated by models that have $A(\phi )=1+\beta (\phi _0)\phi /{M_\mathrm{{pl}}}+\cdots$ and the factor of $-1$ is used to subtract the matter density from the chameleon energy density in order to avoid double counting in cosmology. [The equation of motion (2.7) which governs the dynamics is unchanged by including such a factor]. Since we do not consider cosmology here we will not include this factor.

4 Chameleon models were the first example of screening mechanism that screens using this effect. The scalar *blending in with its environment* inspired the name.

5 Note that point particles do satisfy the equivalence principle because every matter species appearing in the action (2.1) is universally coupled to the Jordan frame metric and, thus, follow the same geodesics. The motion of extended objects is governed by energy-momentum conservation and it is here that the difference arises. See Hui et al. (2009) for an extended discussion of this.

6 Note that our conventions differ from theirs. They use tildes to refer to Einstein frame quantities, whereas we use them to refer to Jordan frame quantities and their function $\varOmega (\phi )$ is related to our coupling function via $\varOmega (\phi )=A^{-1}(\phi )$.

7 Technically one does have an $\mathcal {O}(1)$ contribution to $k(\phi )\approx 1+6{M_\mathrm{{pl}}}^2/M^2$ which can be $\gg 1$ for some values of *M* considered here. In fact, it should be the canonically normalized field, $\varphi =\sqrt{k(\phi )}\phi \sim \mathcal {O}(v^2/c^2)$ (at this order), which is why we can neglect this contribution.

8 Atoms are neutral as well but one advantage of neutrons is that their polarizability is 15 orders of magnitude smaller, making van der Waals forces less of a background. We are grateful to Tobias Jenke for pointing this out to us.

9 We thank Tobias Jenke for providing us with the numeric values.

10 Technically, this is only the case when , corresponding to parameters where the hydrogen burning shell becomes unscreened. When this happens, the core temperature increases at a faster rate leading to a reduction of the tip luminosity because the star has less time time to ascend the RGB. We will see shortly that $\chi >10^{-6}$ can be ruled out by other, independent means and so we will not dwell on this too much here.

11 This has the result that small compressions result in an increased opacity that in turn causes an increase in the energy absorbed. The energy dammed up by this compression drives the pulsations. This is known as the $\kappa$-mechanism.

12 Metallicity and other corrections produce a positive $\varDelta d/d$, which makes the constraints even stronger.

13 This assumes that the gas entirely supported by thermal pressure. In practice, one expects a small amount of non-thermal pressure but N-body simulations of chameleon theories have shown this to be negligible (Wilcox et al. 2016).
